# Supplementary material for: Genetic markers for knee osteoarthritis presence are not associated with disease progression - data from the IMI-APPROACH cohort
Source: PLoS One. 2025 Jun 24;20(6):e0325819. doi: 10.1371/journal.pone.0325819 (PMC12186935; doi:10.1371/journal.pone.0325819)
Supplement: S1 Fig — A set of 54848 autosomal LD-pruned SNPs with MAF > 40% was used to perform the principal component analysis. Three sample populations from the 1000 Genome project were used to compare to the IMI-APPROACH samples [22]. In black = IMI-APPROACH samples, red = 60 samples with European ancestry (CEU), blue = 60 samples with African ancestry (Yoruba, YRI), and green = 90 samples with East Asian ancestry (Japanese/Chinese, JPT)). (DOCX) [file pone.0325819.s001.docx]

**Supplementary Figure S1**

**
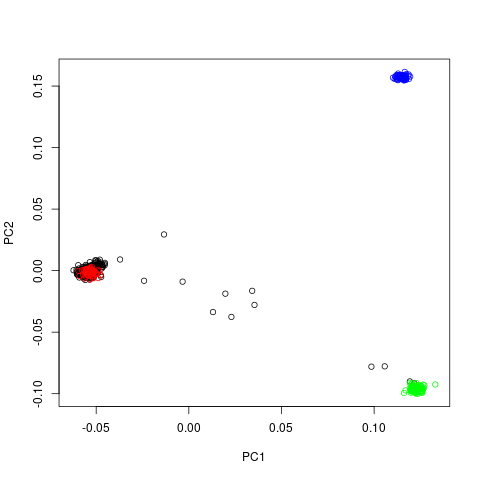
**

**Fig S1. Principal component analysis of the IMI-APPROACH samples against three sample populations from the 1000 Genome project.**

A set of 54848 autosomal LD-pruned SNPs with MAF > 40% was used to perform the principal component analysis. Three sample populations from the 1000 Genome project were compared to the IMI-APPROACH samples [22]. In black = IMI-APPROACH samples, red = 60 samples with European ancestry (CEU), blue = 60 samples with African ancestry (Yoruba, YRI), and green = 90 samples with East Asian ancestry (Japanese/Chinese, JPT)). All IMI-APPROACH samples not clustered with the CEU reference population (red cluster) were excluded from the analysis (n = 13).
