# Supplementary material for: Genetic markers for knee osteoarthritis presence are not associated with disease progression - data from the IMI-APPROACH cohort
Source: PLoS One. 2025 Jun 24;20(6):e0325819. doi: 10.1371/journal.pone.0325819 (PMC12186935; doi:10.1371/journal.pone.0325819)
Supplement: S2 Fig — Manhattan plots showing the -log10(P) values of all ~ 1,5 million SNPs. The x-labels 1–22 represent chromosomes 1–22, 23 is the X chromosome, 24 is the Y chromosome, 25 is the pseud-autosomal region of X, and 26 is mitochondrial chromosomes (0 are unplaced SNPs). The red line represents the genome-wide significance threshold (P < 5 × 10^−8^). Green dots indicate SNPs that were associated with knee OA or OA in general in previous GWAS (meta-analysis) [7,9]. The Q-Q plot shows the genome-wide –log10(P) values of the association analysis, where the red line corresponds to the null hypothesis. (A) Manhattan plot increasing pain (KOOS score increase of ≥5 or ≥ 10 per year with a minimum score of ≥ 35 or ≥ 40 at 2 years). (B) Q-Q plot increasing pain. (C) Manhattan plot stable high pain (KOOS score of ≥40 over 2 years). (D) Q-Q plot stable high pain. (E) Manhattan plot presence of decrease in minJSW (minimum decrease of ≤ 0.3 mm/year at 2 years). (F) Q-Q plot presence of decrease in minJSW. (G) Manhattan plot change in pain (change in KOOS score). (H) Q-Q plot change in pain. (I) Manhattan plot presence of radiographic OA (Kellgren Lawrence (KL) ≥ 2 at 2 years). (J) Q-Q plot radiographic OA. (DOCX) [file pone.0325819.s002.docx]

**Supplementary Figure S2**
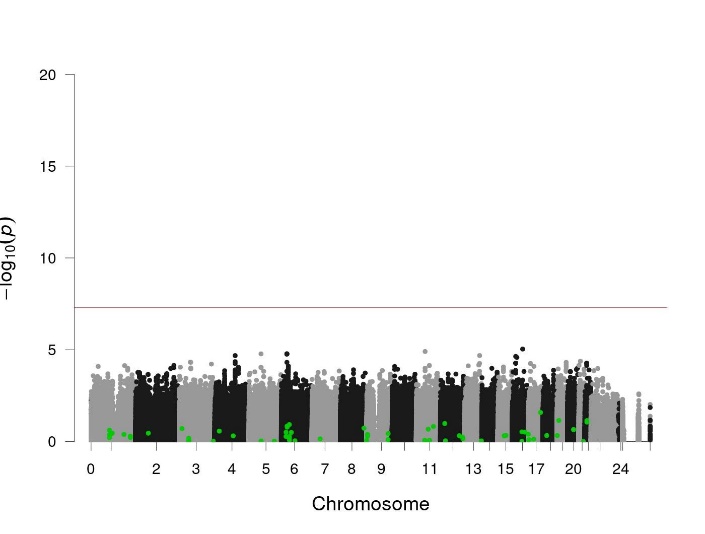

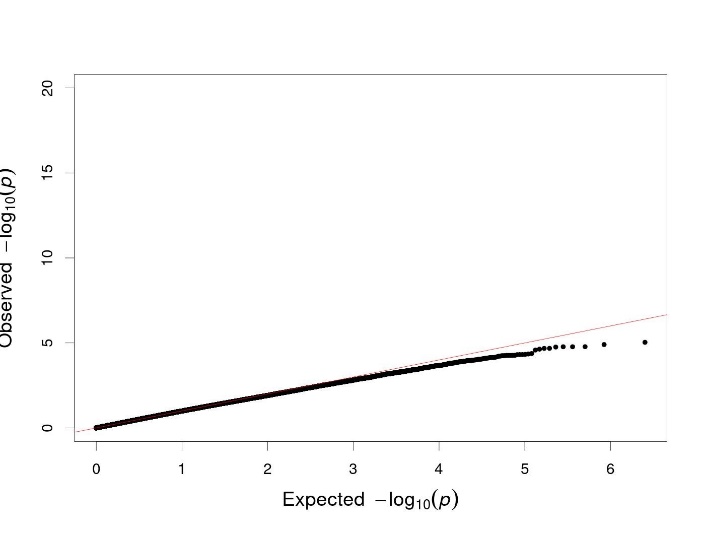


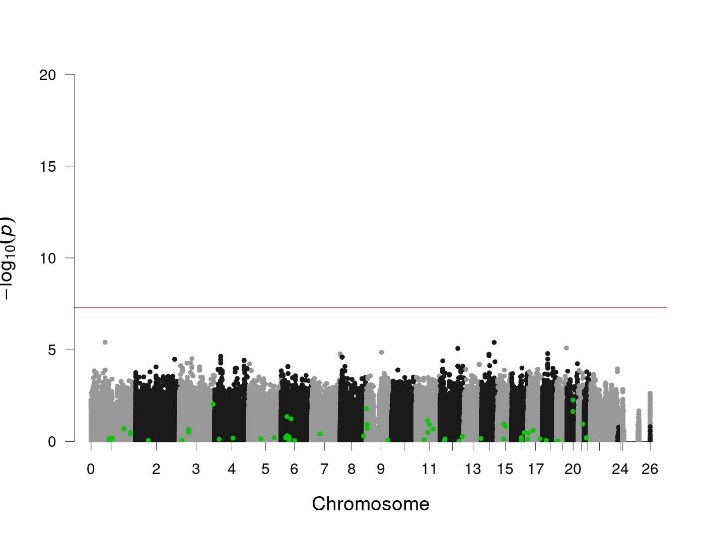

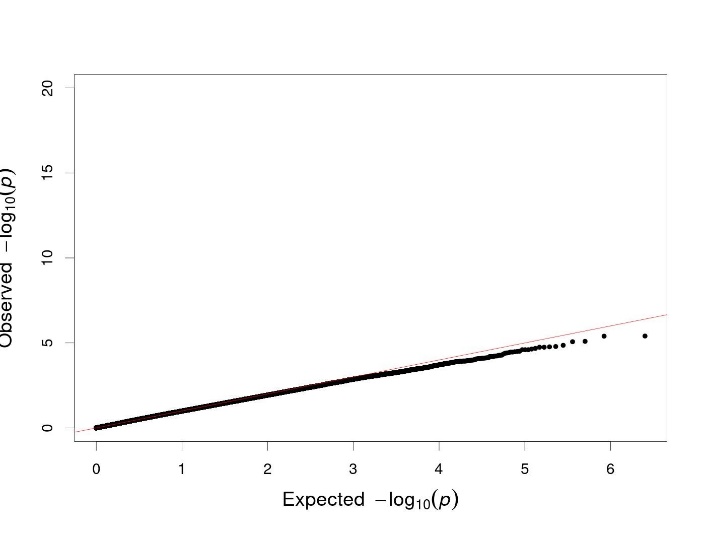

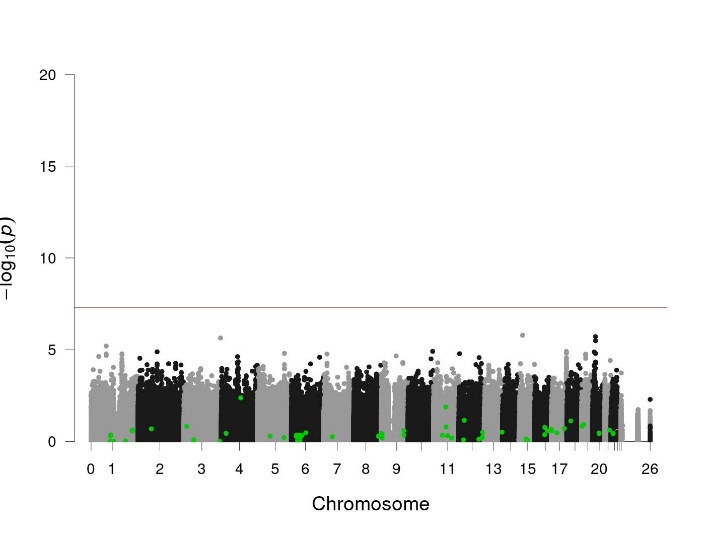

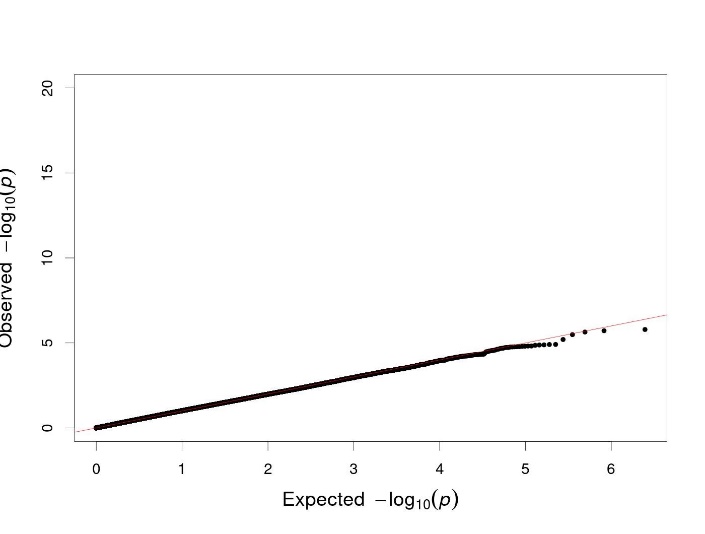

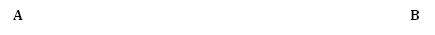

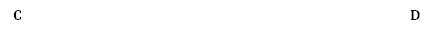

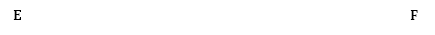

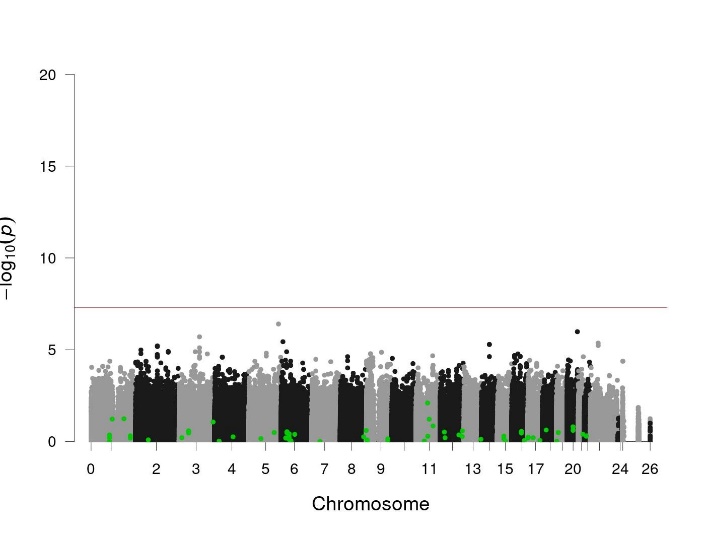

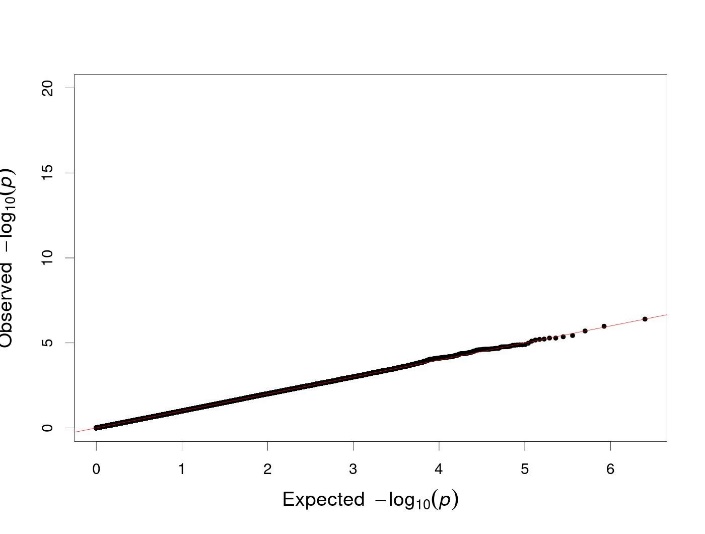

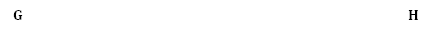

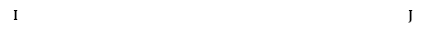

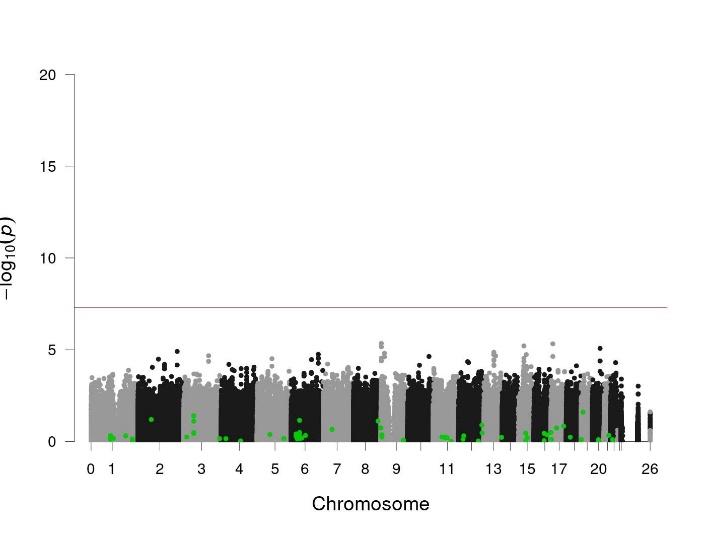

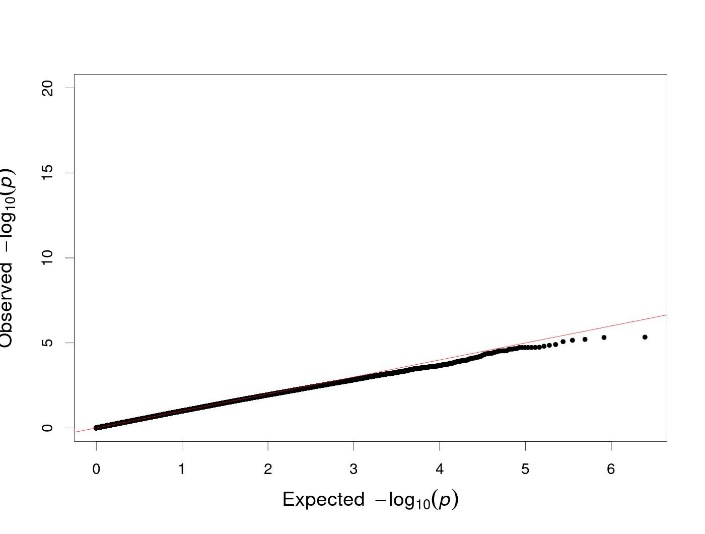


**Fig S2.** **Validation of genetic markers for risk of OA for five knee OA progression outcomes.**

Manhattan plots showing the -log10(P) values of all ~1,5 million SNPs. The x-labels 1-22 represent chromosomes 1-22, 23 is the X chromosome, 24 is the Y chromosome, 25 is the pseud-autosomal region of X, and 26 is mitochondrial chromosomes (0 are unplaced SNPs). The red line represents the genome-wide significance threshold (*P<* 5 × 10^−8^). Green dots indicate SNPs that were associated with knee OA or OA in general in previous GWAS (meta-analysis) [7,9]. The Q-Q plot shows the genome-wide –log10(P) values of the association analysis, where the red line corresponds to the null hypothesis. (A) Manhattan plot increasing pain (KOOS score increase of ≥5 or ≥ 10 per year with a minimum score of ≥ 35 or ≥ 40 at 2 years). (B) Q-Q plot increasing pain. (C) Manhattan plot stable high pain (KOOS score of ≥40 over 2 years). (D) Q-Q plot stable high pain. (E) Manhattan plot presence of decrease in minJSW (minimum decrease of ≤ 0.3 mm/year at 2 years). (F) Q-Q plot presence of decrease in minJSW. (G) Manhattan plot change in pain (change in KOOS score). (H) Q-Q plot change in pain. (I) Manhattan plot presence of radiographic OA (Kellgren Lawrence (KL) ≥ 2 at 2 years). (J) Q-Q plot radiographic OA.
