# Supplementary material for: Genetic markers for knee osteoarthritis presence are not associated with disease progression - data from the IMI-APPROACH cohort
Source: PLoS One. 2025 Jun 24;20(6):e0325819. doi: 10.1371/journal.pone.0325819 (PMC12186935; doi:10.1371/journal.pone.0325819)
Supplement: S3 Fig — Association with minJSW decrease (-log10(P)) of SNPs is plotted against the genomic position. The colour represents the pairwise correlation coefficient (Linkage disequilibrium pattern) of each SNP with the most significant SNP (shown as the purple square, in Fig A. rs73146904 and Fig B. rs2549732). (A) The regional plot of rs73146904 shows that several SNPs lay within the near region of PLCL2. (B) Regional plot of rs2549732 shows that several SNPs lay within the near region of CDYL2. (DOCX) [file pone.0325819.s003.docx]

**Supplementary Figure S3**

**Fig S3. Regional plots of rs73146904 and rs2549732 based on the Genome Reference Consortium Human Build 37.**


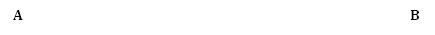

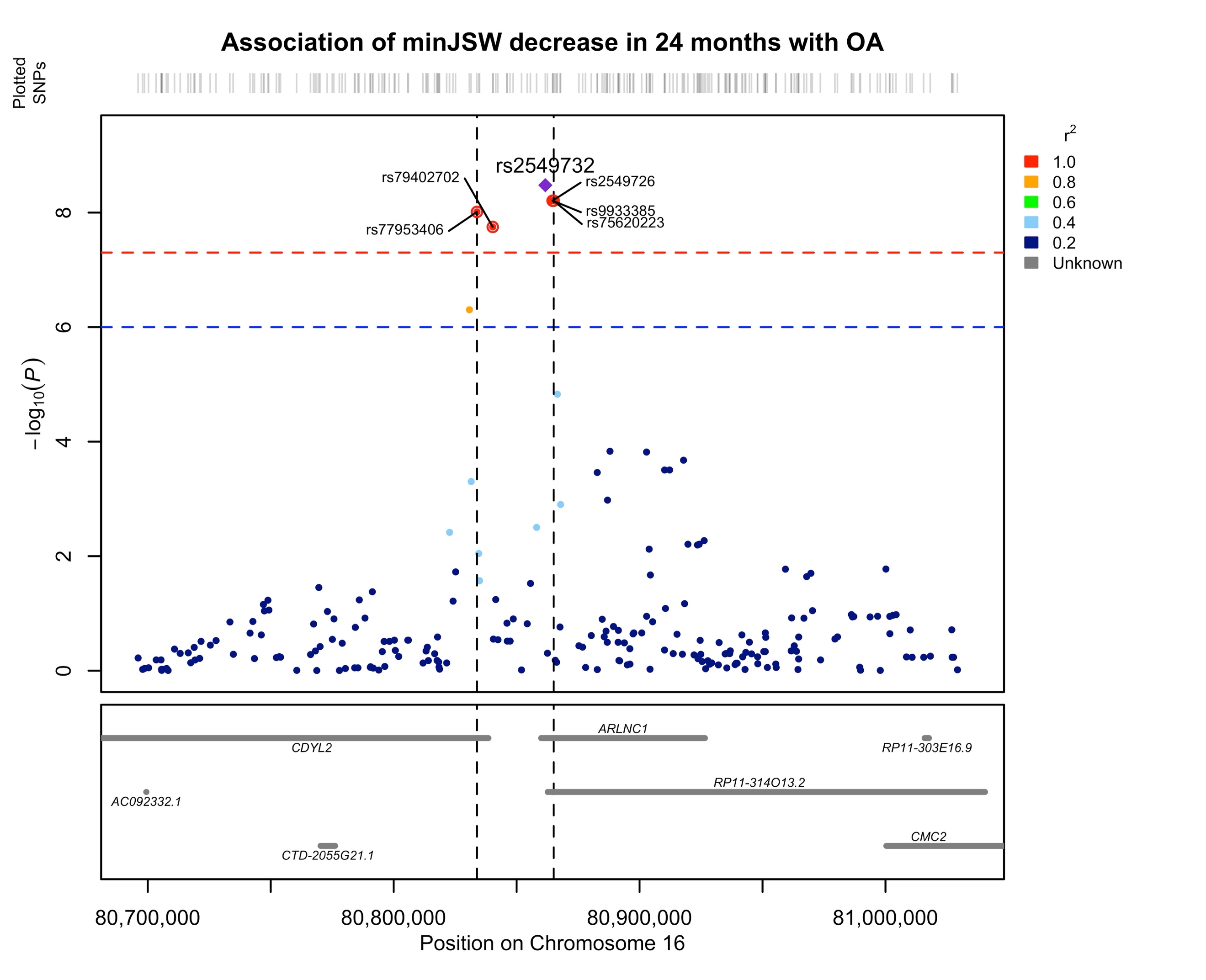

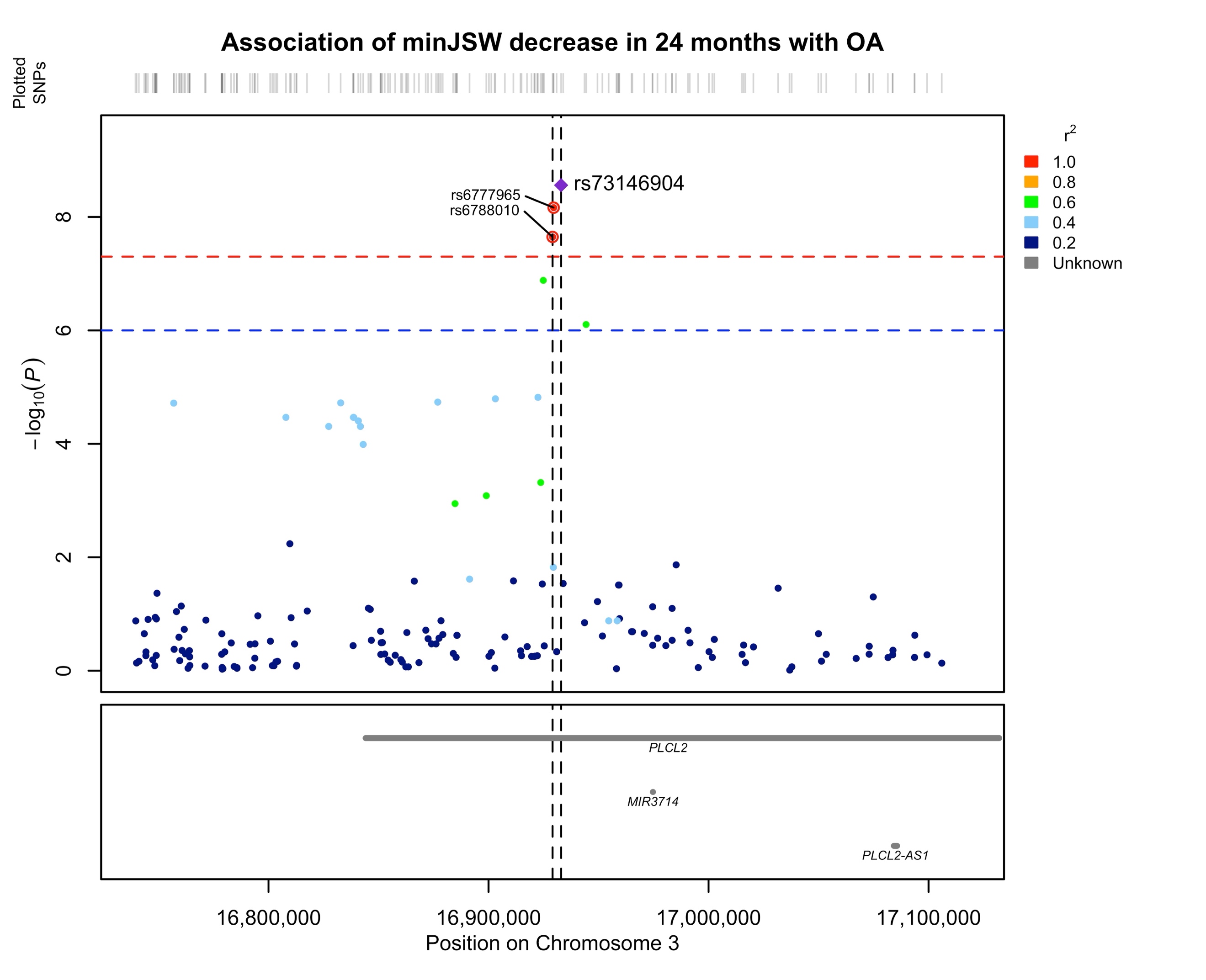


Association with minJSW decrease (-log10(P)) of SNPs is plotted against the genomic position. The colour represents the pairwise correlation coefficient (Linkage disequilibrium pattern) of each SNP with the most significant SNP (shown as the purple square, in Fig A. rs73146904 and Fig B. rs2549732). (A) The regional plot of rs73146904 shows that several SNPs lay within the near region of *PLCL2*. (B) Regional plot of rs2549732 shows that several SNPs lay within the near region of *CDYL2*.
