# Supplementary material for: Genetic markers for knee osteoarthritis presence are not associated with disease progression - data from the IMI-APPROACH cohort
Source: PLoS One. 2025 Jun 24;20(6):e0325819. doi: 10.1371/journal.pone.0325819 (PMC12186935; doi:10.1371/journal.pone.0325819)
Supplement: S4 Fig — Comparison of gene expression levels at baseline is shown of PLCL2 and CDYL2 of the patients split into groups based on their genotype. None of the plots show significant up- or down-regulation. The number of patients within a group is noted below the boxplot. The patients with two minor alleles are underrepresented. The black lines indicate the median values. The following SNPs are located near PLCL2 and shown in the plots: (A) rs6442653 versus PLCL2. (B) rs6788010 versus PLCL2. (C) rs6777965 versus PLCL2. (D) rs73146904 versus PLCL2. (E) rs77953406 versus CDYL2. (F) rs79402702 versus CDYL2. (G) rs2549732 versus CDYL2. (H) rs2549726 versus CDYL2. (I) rs9933385 versus CDYL2. (J) rs75620223 versus CDYL2. (DOCX) [file pone.0325819.s004.docx]

**Supplementary Figure S4**


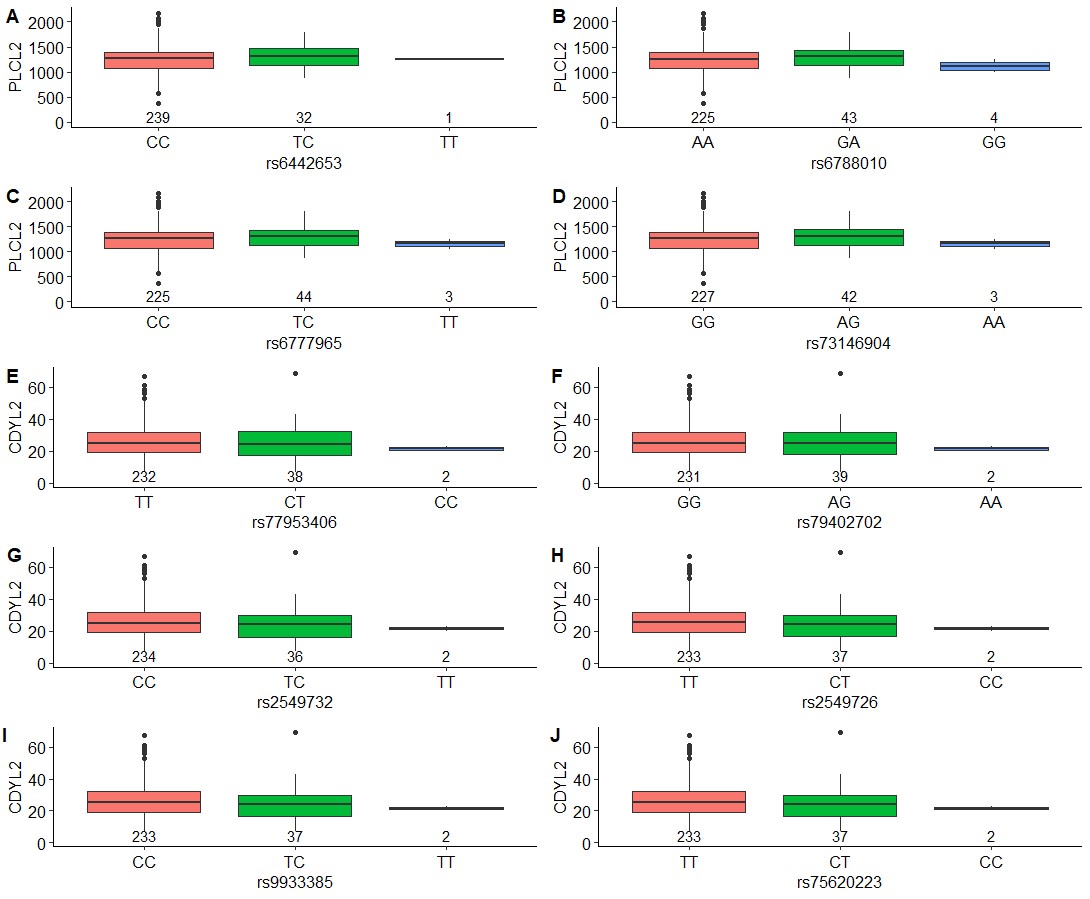
**Fig S4**. **Comparison of expression levels of *PLCL2* and *CDYL2* associated with ten significant SNPs.**

Comparison of gene expression levels at baseline is shown of *PLCL2* and *CDYL2* of the patients split into groups based on their genotype. None of the plots show significant up- or down-regulation. The number of patients within a group is noted below the boxplot. The patients with two minor alleles are underrepresented. The black lines indicate the median values. The following SNPs are located near *PLCL2* and shown in the plots: (A) rs6442653 versus *PLCL2*. (B) rs6788010 versus *PLCL2*. (C) rs6777965 versus *PLCL2*. (D) rs73146904 versus *PLCL2*. (E) rs77953406 versus *CDYL2*. (F) rs79402702 versus *CDYL2*. (G) rs2549732 versus *CDYL2*. (H) rs2549726 versus *CDYL2*. (I) rs9933385 versus *CDYL2*. (J) rs75620223 versus *CDYL2*.
