# Supplementary material for: Genetic markers for knee osteoarthritis presence are not associated with disease progression - data from the IMI-APPROACH cohort
Source: PLoS One. 2025 Jun 24;20(6):e0325819. doi: 10.1371/journal.pone.0325819 (PMC12186935; doi:10.1371/journal.pone.0325819)
Supplement: S5 Fig — The violin plots of the expression quantitative trait loci (eQTLs) of muscle-skeletal tissue show up- and down-expression of the gene (normalised expression) based on the alleles for six SNPs located near CDYL2. The white line in the box plot (shown within the black boxes) represents the median value of the expression. All eQTLs show a significant effect of the genotype on the normalised expression of the gene (all p-values are below the gene p-value threshold of 0.00012). (DOCX) [file pone.0325819.s005.docx]

**Supplementary Figure S5**

**Fig S5. Expression quantitative trait loci in muscle-skeletal tissue, associated with six significant SNPs located near *CDYL2*.**


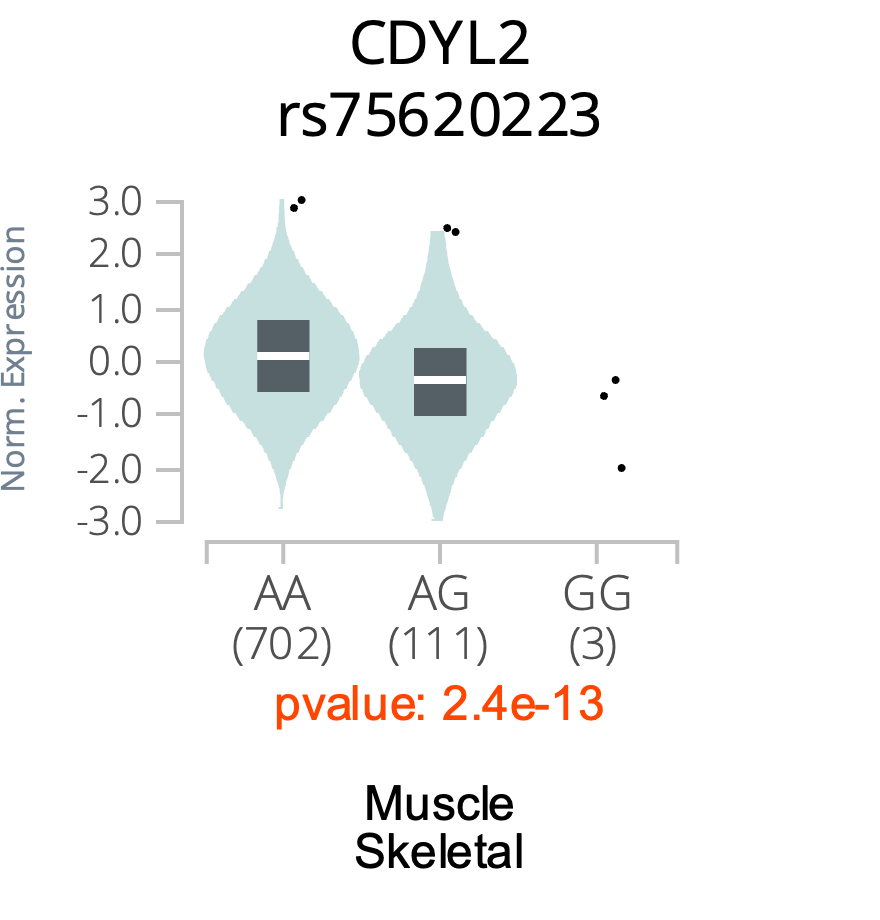

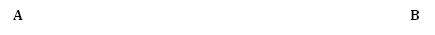

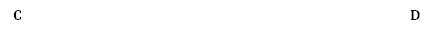

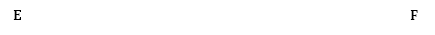

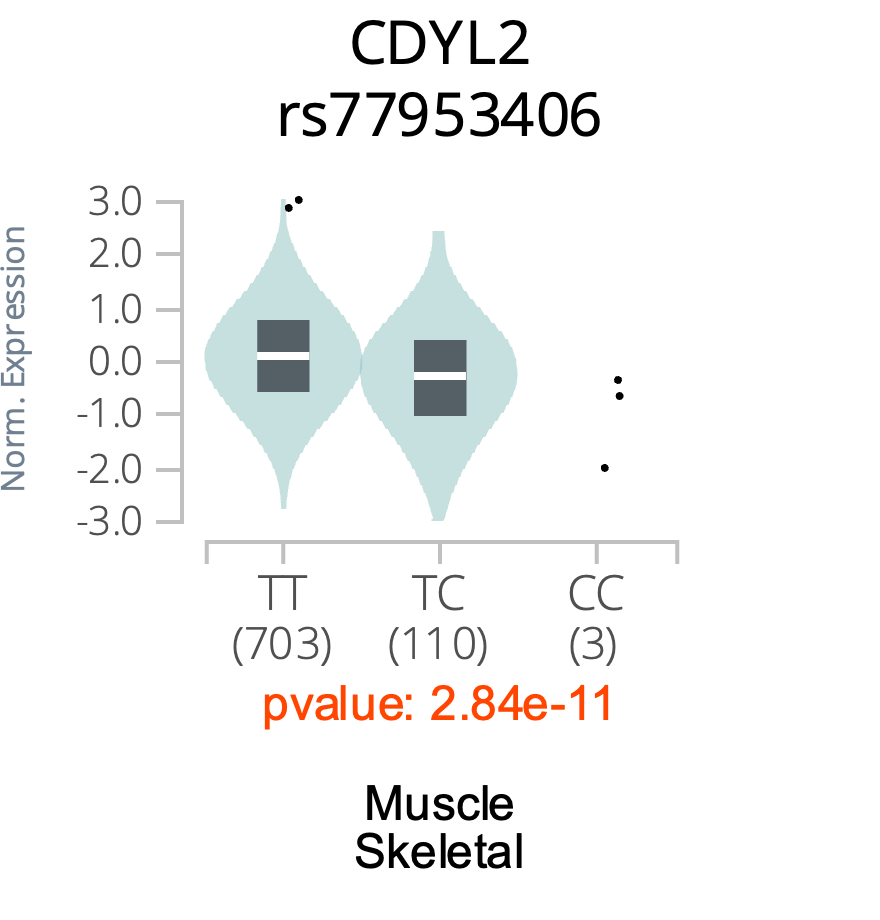

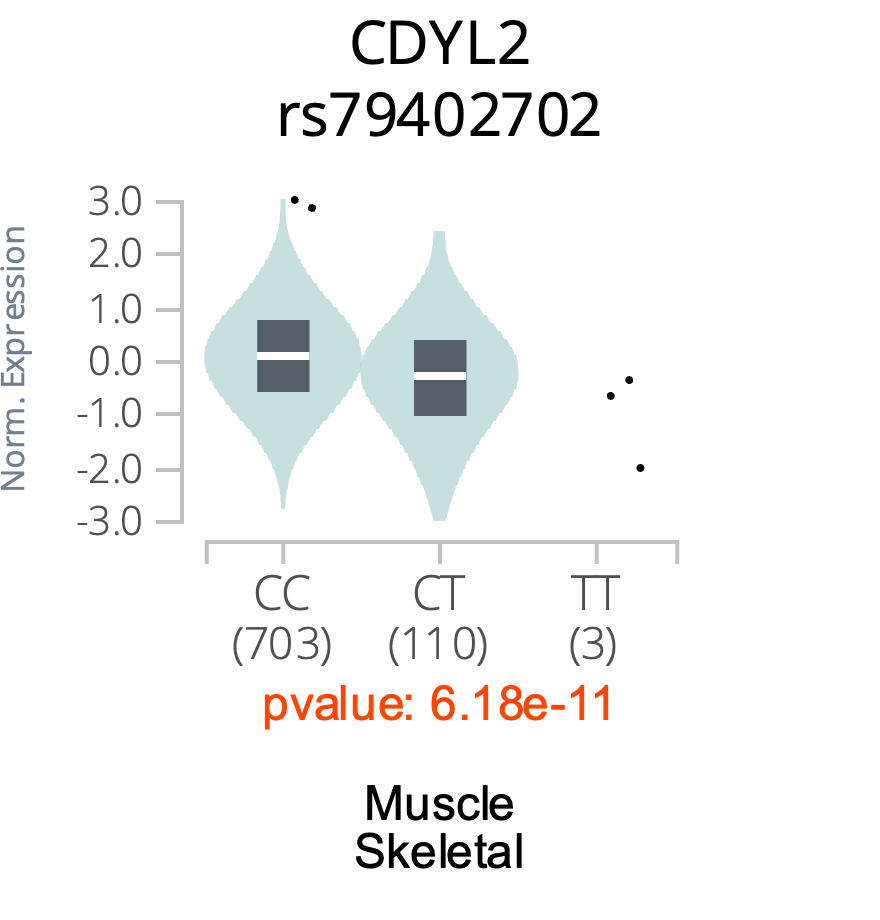

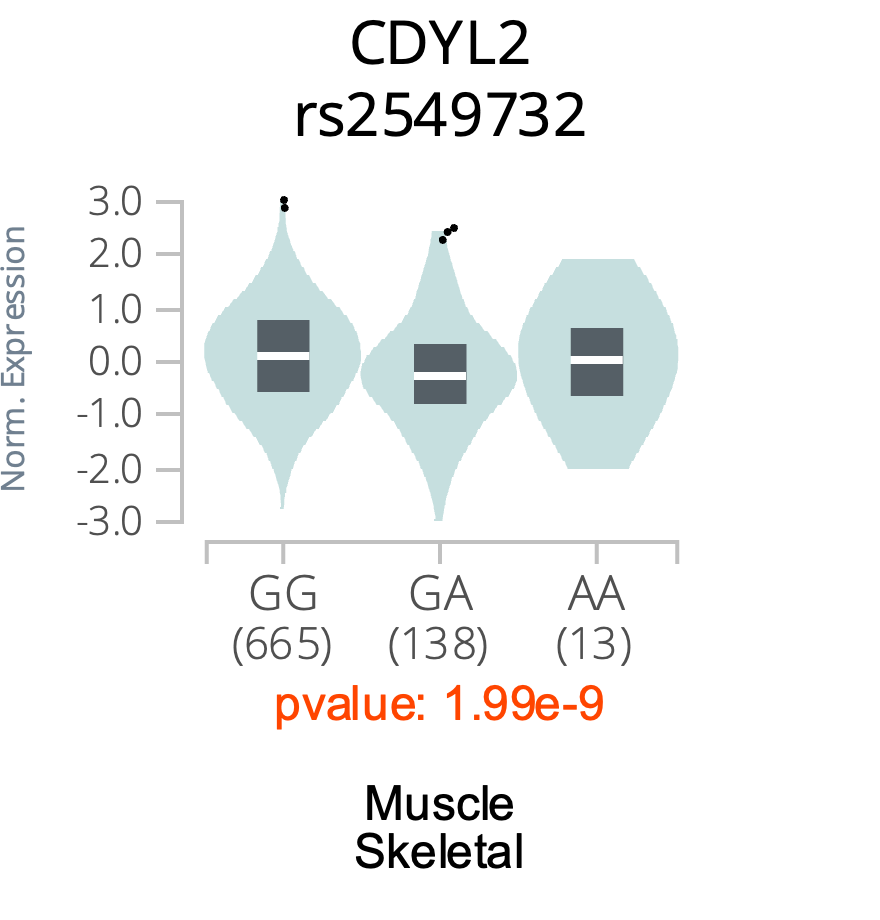

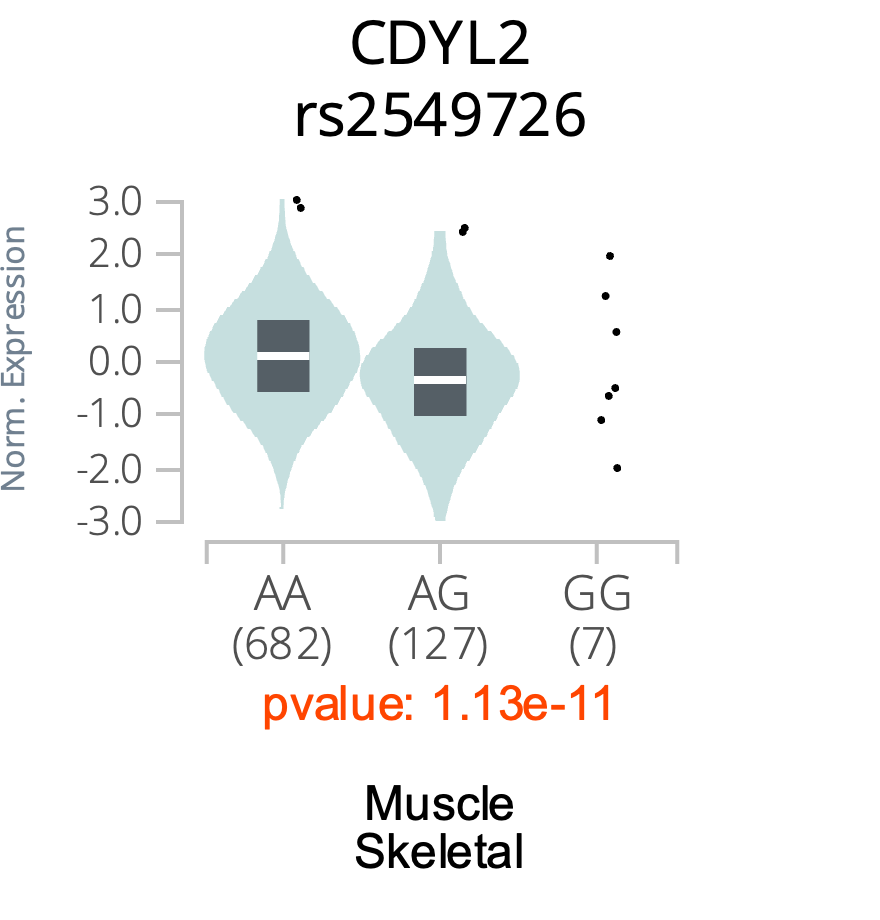

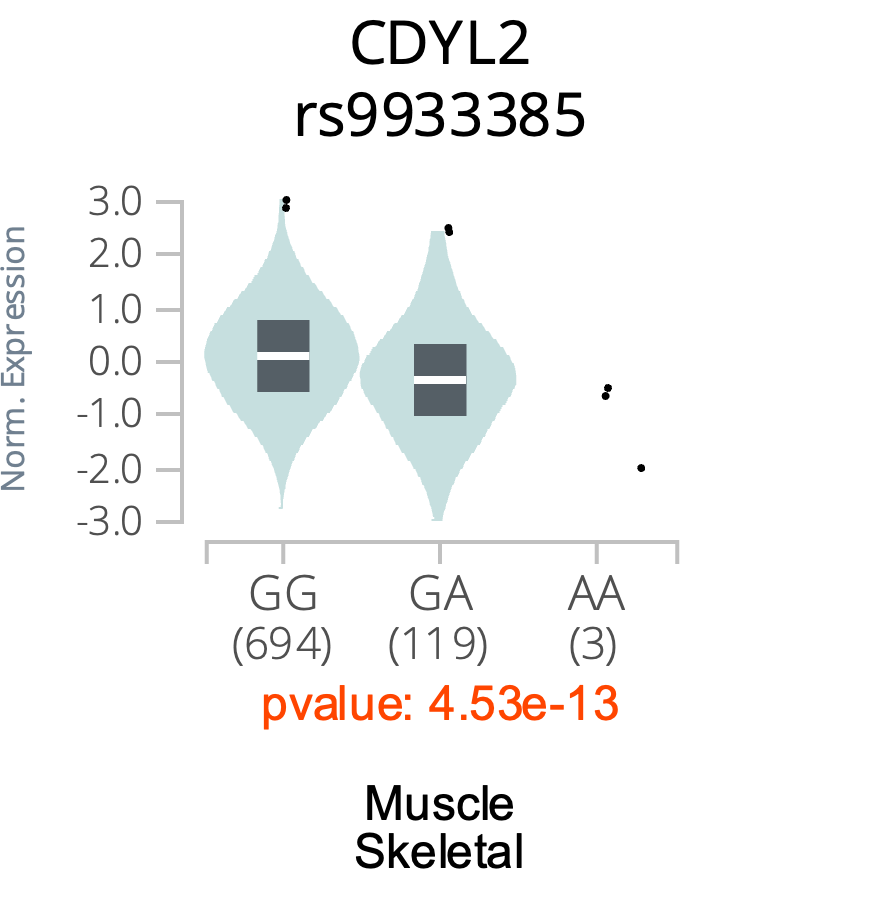


The violin plots of the expression quantitative trait loci (eQTLs) of muscle-skeletal tissue show up- and down-expression of the gene (normalised expression) based on the alleles for six SNPs located near *CDYL2*. The white line in the box plot (shown within the black boxes) represents the median value of the expression. All eQTLs show a significant effect of the genotype on the normalised expression of the gene (all p-values are below the gene p-value threshold of 0.00012).
