# Supplementary material for: Genetic markers for knee osteoarthritis presence are not associated with disease progression - data from the IMI-APPROACH cohort
Source: PLoS One. 2025 Jun 24;20(6):e0325819. doi: 10.1371/journal.pone.0325819 (PMC12186935; doi:10.1371/journal.pone.0325819)
Supplement: S6 Fig — The minJSW decrease is in mm over 2 years. Each dot represents a patient. (A) Change in minJSW in mm at 2 years versus the normalised expression of PLCL2 at baseline. The x-coefficient (representing PLCL2) is not significant (P > 0.05). (B) Change in minJSW in mm at 2 years versus the normalised expression of CDYL2 at baseline. The x-coefficient (representing CDYL2) is 0.01 and is significant: P = 0.044. (DOCX) [file pone.0325819.s006.docx]

**Supplementary Figure S6**


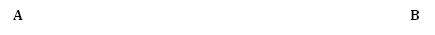

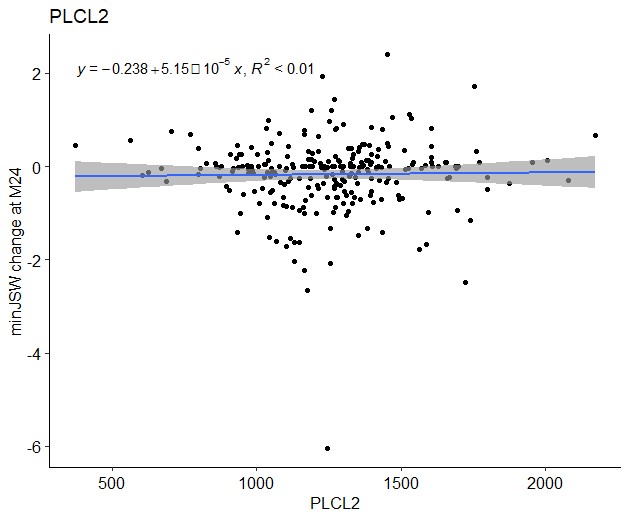

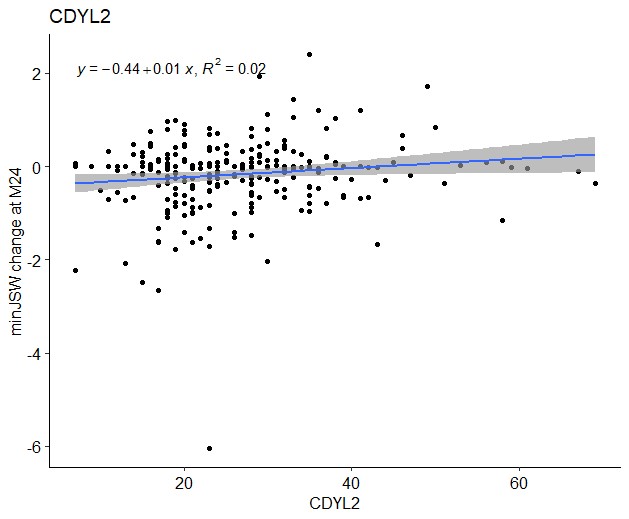


**Fig S6. Scatter-plot of minJSW decrease versus expression of *PLCL2* and *CDYL2*.**

The minJSW decrease is in mm over 2 years. Each dot represents a patient. (A) Change in minJSW in mm at 2 years versus the normalised expression of *PLCL2* at baseline. The x-coefficient (representing *PLCL2*) is not significant (P>0.05) (B) Change in minJSW in mm at 2 years versus the normalised expression of *CDYL2* at baseline. The x-coefficient (representing *CDYL2*) is 0.01 and is significant: P = 0.044.
