# Supplementary material for: Genetic markers for knee osteoarthritis presence are not associated with disease progression - data from the IMI-APPROACH cohort
Source: PLoS One. 2025 Jun 24;20(6):e0325819. doi: 10.1371/journal.pone.0325819 (PMC12186935; doi:10.1371/journal.pone.0325819)
Supplement: S7 Fig — Protein-protein interaction network, created with STRING-db, between the protein-coding genes associated with the identified significant SNPs (in red). No known direct associations are shown. (DOCX) [file pone.0325819.s007.docx]

**Supplementary Figure S7**


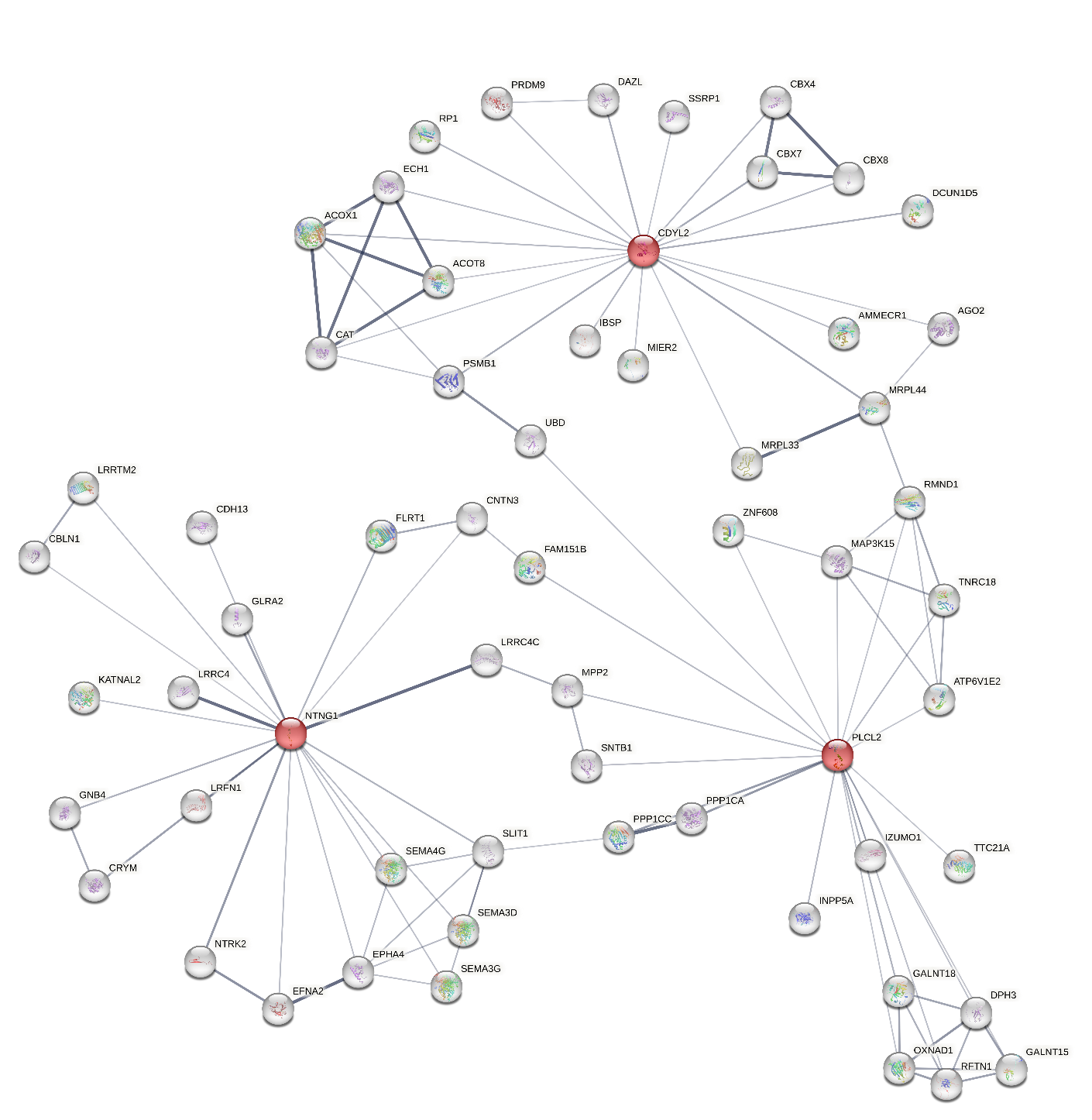


**Fig S7.** **Protein-protein interaction network between the protein-coding genes near significant SNPs.**
Protein-protein interaction network, created with STRING-db, between the protein-coding genes associated with the identified significant SNPs (in red). No known direct associations are shown.
