# Supplementary material for: Genetic markers for knee osteoarthritis presence are not associated with disease progression - data from the IMI-APPROACH cohort
Source: PLoS One. 2025 Jun 24;20(6):e0325819. doi: 10.1371/journal.pone.0325819 (PMC12186935; doi:10.1371/journal.pone.0325819)
Supplement: S8 Fig — The network, created with STRING-db, includes both protein-coding genes associated with the 19 newly identified significant SNPs (in red), as well as the protein-coding genes as reported along the SNPs identified to be associated with OA in previous GWAS. No direct links are found between genes related to the newly found SNPs and genes near previously established SNPs. (DOCX) [file pone.0325819.s008.docx]

**Supplementary Figure S8**


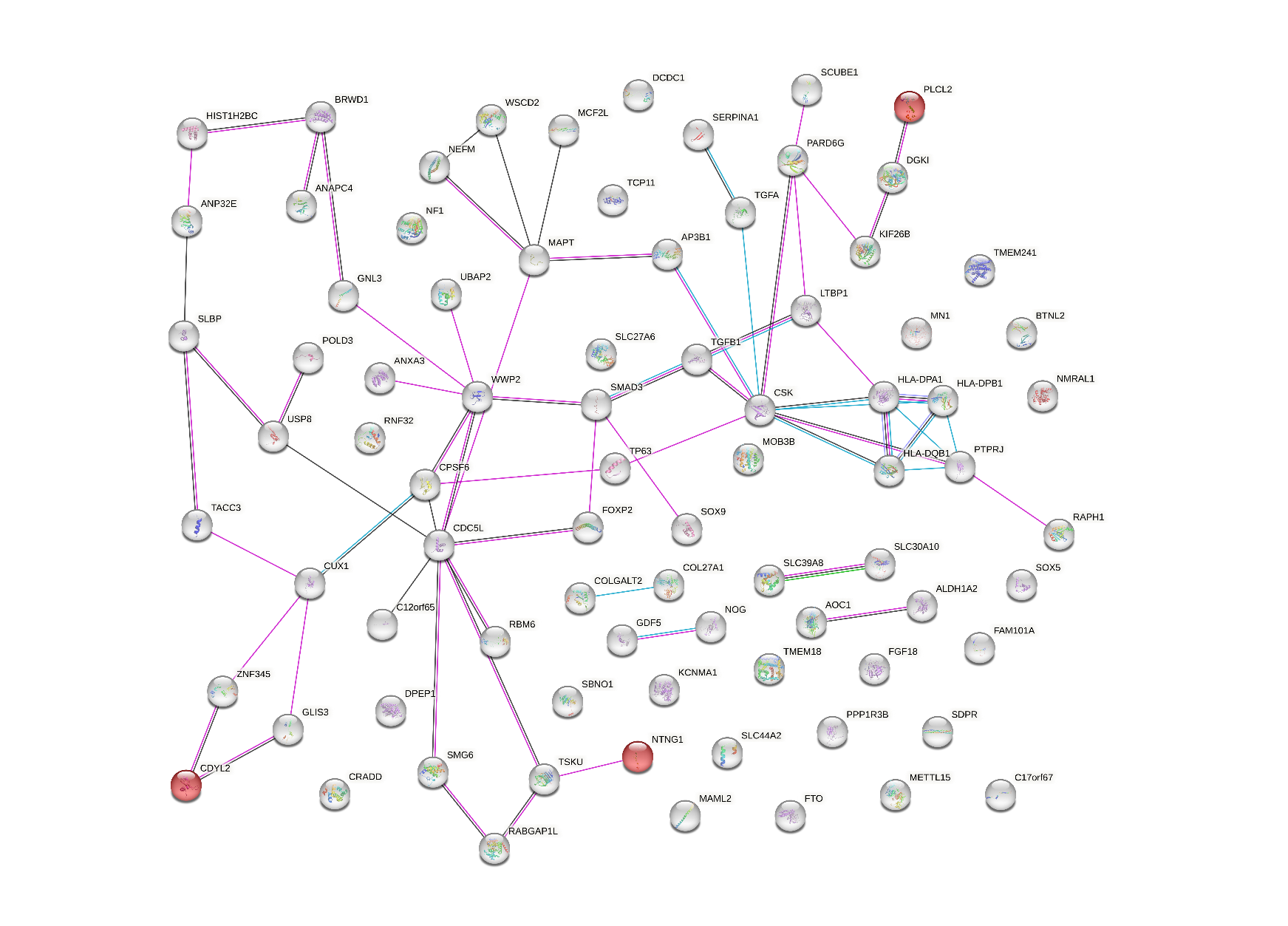


**Fig S8.** **Protein-protein interaction network of proteins near known and newly identified SNPS.**The network, created with STRING-db, includes both protein-coding genes associated with the 19 newly identified significant SNPs (in red), as well as the protein-coding genes as reported along the SNPs identified to be associated to OA in previous GWAS. No direct links are found between genes related to the newly found SNPs and genes near previously established SNPs.
