# Supplementary material for: Genetic markers for knee osteoarthritis presence are not associated with disease progression - data from the IMI-APPROACH cohort
Source: PLoS One. 2025 Jun 24;20(6):e0325819. doi: 10.1371/journal.pone.0325819 (PMC12186935; doi:10.1371/journal.pone.0325819)
Supplement: S1 Table — The PRS is calculated using 30 OA presence SNPs. The associations of the PRS with OA progression outcomes, as was found with linear and logistic regression, are summarised in this table. The reduction in minJSW in mm over 2 years and change in KOOS score were used in linear regression. The presence of increasing pain, stable high pain, radiographic OA, and minJSW decrease of ≥ 0.3 mm/year were evaluated using logistic regression. (DOCX) [file pone.0325819.s009.docx]

**Supplementary Table S1**

**Table S1. Polygenic risk score and OA progression association**

| **Outcome** | **Beta [95% CI]** | **p-value** | **Adjusted R2** |
| --- | --- | --- | --- |
| minJSW decrease in mm over 2 years | 1.403 [-17.084 – 14.278] | 0.862 | 0.00 |
| KOOS change in pain | -6.939 [-371.357 – 357.479] | 0.970 | 0.00 |
| KOOS increasing pain | 11.007 [-47.500 – 69.514] | 0.712 | 0.00 |
| KOOS constant high pain | -23.821 [-77.298 – 29.656] | 0.383 | 0.00 |
| minJSW decrease of 0.3 mm /year | 17.545 [-42.716 – 77.806] | 0.568 | 0.00 |
| Radiographic OA | 7.619 [-33.182 – 48.421] | 0.714 | 0.00 |

The PRS is calculated using 30 OA presence SNPs. The associations of the PRS with OA progression outcomes, as was found with linear and logistic regression, is summarised in this table. The reduction in minJSW in mm over 2 years and change in KOOS score were used in linear regression. The presence of increasing pain, stable high pain, radiographic OA, and minJSW decrease of ≥ 0.3 mm/year were evaluated using logistic regression.
