# Supplementary material for: Genetic markers for knee osteoarthritis presence are not associated with disease progression - data from the IMI-APPROACH cohort
Source: PLoS One. 2025 Jun 24;20(6):e0325819. doi: 10.1371/journal.pone.0325819 (PMC12186935; doi:10.1371/journal.pone.0325819)
Supplement: S1 File — (DOCX) [file pone.0325819.s010.docx]

**Supplementary Information S1**

# RNA-sequencing data.

RNA was extracted from whole blood samples collected on Paxgene tubes obtained at baseline. Both protein-coding and noncoding transcripts were sequenced on an Illumina platform for 286 patients and 4 controls. A Strand-specific RNA library preparation with rRNA and globin depletion was performed. Some samples required DNase treatment. Both protein-coding and non-coding transcripts were sequenced by NGS (Next-Generation sequencing) on an Illumina platform with a paired-end 150 bp sequencing strategy (Genewiz). Servier performed quality control of RNA-seq experiment and pre-processing of data in order to quantify gene expression.

Quality control and data pre-processing has been completed, confirming at least 20M reads and RIN>6 for most samples. The data was normalized to avoid impact of some highly repeated genes on quantitative analyses of gene expression.
