# Supplementary material for: Genetic markers for knee osteoarthritis presence are not associated with disease progression - data from the IMI-APPROACH cohort
Source: PLoS One. 2025 Jun 24;20(6):e0325819. doi: 10.1371/journal.pone.0325819 (PMC12186935; doi:10.1371/journal.pone.0325819)
Supplement: Attachment — Submitted filename: Response to Reviewers.docx [file pone.0325819.s012.docx]

**We thank the editor and reviewers for the helpful comments by which we feel the manuscript has improved. Please find an itemized response to all questions below (in blue). Hopefully, we answered all questions and addressed all comments to the intention and satisfaction of the reviewers.**

**Responses to questions of the editor**

1. Please ensure that your manuscript meets PLOS ONE's style requirements, including those for file naming. The PLOS ONE style templates can be found at

**Response**:	We have checked the PLOS ONE style requirements and made adjustments when necessary.

2. Thank you for stating the following financial disclosure:

“The research leading to these results has received support from the Innovative Medicines Initiative Joint Undertaking under Grant Agreement no 115770, resources of which are composed of financial contribution from the European Union’s Seventh Framework Programme (FP7/2007–2013) and EFPIA companies’ in kind contribution. See [www.imi.europa.eu](http://www.imi.europa.eu) and <http://www.approachproject.eu>. This manuscript reflects the views of the authors and neither IMI nor the European Union and EFPIA are liable for any use that may be made of the information contained herein.”

Please state what role the funders took in the study.  If the funders had no role, please state: "The funders had no role in study design, data collection and analysis, decision to publish, or preparation of the manuscript."

If this statement is not correct you must amend it as needed.

Please include this amended Role of Funder statement in your cover letter; we will change the online submission form on your behalf.

**Response**:	The funders had no role in study design, data collection and analysis, decision to publish, or preparation of the manuscript. We have included this in the cover letter.

3. Thank you for stating the following in the Acknowledgments Section of your manuscript:

“The research leading to these results has received support from the Innovative Medicines Initiative Joint Undertaking under Grant Agreement no 115770, resources of which are composed of financial contribution from the European Union’s Seventh Framework Programme (FP7/2007–2013) and EFPIA companies’ in kind contribution. See [www.imi.europa.eu](http://www.imi.europa.eu) and [http://www.approachproject.eu.”](http://www.approachproject.eu.xn--ivg)

We note that you have provided funding information that is currently declared in your Funding Statement. However, funding information should not appear in the Acknowledgments section or other areas of your manuscript. We will only publish funding information present in the Funding Statement section of the online submission form.

Please remove any funding-related text from the manuscript and let us know how you would like to update your Funding Statement. Currently, your Funding Statement reads as follows:

“The research leading to these results has received support from the Innovative Medicines Initiative Joint Undertaking under Grant Agreement no 115770, resources of which are composed of financial contribution from the European Union’s Seventh Framework Programme (FP7/2007–2013) and EFPIA companies’ in kind contribution. See [www.imi.europa.eu](http://www.imi.europa.eu) and <http://www.approachproject.eu>. This manuscript reflects the views of the authors and neither IMI nor the European Union and EFPIA are liable for any use that may be made of the information contained herein.”

Please include your amended statements within your cover letter; we will change the online submission form on your behalf.

**Response**:	We have included our amended statements in the cover letter.

4. Thank you for stating the following in the Competing Interests section:

“I have read the journal's policy and the authors of this manuscript have the following competing interests: Outside the current manuscript, PM is employed by Servier and declares no competing interests in relationship with this manuscript; MK reports grants from IMI-APPROACH, grants from Dutch Arthritis Association, royalties from Wolters Kluwer and Springer Verlag, consulting fees (all paid to institution) from Pfizer, Galapagos, CHDR, Novartis and UCB, and MK is a member of the EULAR council; FJB reports payment to the institution from Gedeon Richter Plc., Bristol-Myers Squibb International corporation (BMSIC), Sun Pharma Global FZE, Celgene Corporation, Janssen Cilag International N.V, Janssen Research & Development, Viela Bio, Inc., Astrazeneca AB, UCB BIOSCIENCES GMBH, UCB BIOPHARMA SPRL, AbbVie Deutschland GmbH & Co.KG, Merck KGaA, Amgen, Inc., Novartis Farmacéutica, S.A., Boehringer Ingelheim España, S.A, CSL Behring, LLC, Glaxosmithkline Research & Development Limited, Pfizer Inc, Lilly S.A., Corbus Pharmaceuticals Inc., Biohope Scientific Solutions for Human Health S.L., Centrexion Therapeutics Corp., Sanofi, MEIJI FARMA S.A., Kiniksa Pharmaceuticals, Ltd, Fundación para la Investigación Biomédica Del Hospital Clínico San Carlos, Grünenthal, Galapagos; IKH reports personal fees from Novartis, GSK and Grünenthal; FB reports consulting fees from Grünenthal, GSK, Eli Lilly, Novartis, Pfizer, Servier, honoraria for lectures from Viatris and Pfizer, personal fees from AstraZeneca, Sun Pharma, Nordic Pharma and Nordic Bioscience, and is shareholder of 4Moving Biotech and 4P Pharma. The other authors have declared no conflicts of interest.”

Please confirm that this does not alter your adherence to all PLOS ONE policies on sharing data and materials, by including the following statement: "This does not alter our adherence to  PLOS ONE policies on sharing data and materials.” (as detailed online in our guide for authors <http://journals.plos.org/plosone/s/competing-interests>).  If there are restrictions on sharing of data and/or materials, please state these. Please note that we cannot proceed with consideration of your article until this information has been declared.

Please include your updated Competing Interests statement in your cover letter; we will change the online submission form on your behalf.

**Response**:	We have included our updated Competing Interests statement in the cover letter.

5. In the online submission form, you indicated that your data is available only on request from a third party. Please note that your Data Availability Statement is currently missing the contact details for the third party, such as an email address or a link to where data requests can be made. Please update your statement with the missing information.

**Response**:	We have included contact details where data requests can be made.

6. We note that you have included the phrase “data not shown” in your manuscript. Unfortunately, this does not meet our data sharing requirements. PLOS does not permit references to inaccessible data. We require that authors provide all relevant data within the paper, Supporting Information files, or in an acceptable, public repository. Please add a citation to support this phrase or upload the data that corresponds with these findings to a stable repository (such as Figshare or Dryad) and provide and URLs, DOIs, or accession numbers that may be used to access these data. Or, if the data are not a core part of the research being presented in your study, we ask that you remove the phrase that refers to these data.

**Response**:	We have included results now in the Supplementary Table S1.

**Responses to questions of reviewer #1**

Reviewer #1: Title: Genetic markers for knee osteoarthritis presence are not associated with disease progression - data from the IMI-APPROACH cohort

This study was designed to validate genetic markers involved in the disease progression of knee OA, an important disease. Unfortunately, the authors did not obtain the expected results, and the data showing that newly identified SNPs are associated with knee OA are not strong. As the authors comment, knee OA is a very heterogeneous disease, and studies using a larger number of subjects than the relatively small number in this study may be necessary to identify certain trends. On the other hand, the longitudinal evaluation in this study was conducted over a period of 24 months, and the disease progression of knee OA was used as an outcome, which we believe has some value in this regard. However, the definition of "disease progression of knee OA" and past findings related to the obtained results are not sufficiently presented. In addition, the current conclusions seem to contain a lot of content that is not directly derived from the obtained results. I do not believe that it is appropriate to publish the report, at least in its current form.

**Response**:	Thanks for your feedback. Below we respond to your specific remarks and suggestions.

Major concerns:

1. Conclusions
I believe that only conclusions derived from the results should be stated simply. Especially in the final paragraph of the main text, considerations and assertions are conspicuous. I suppose the message to the reader will become clearer and more appropriate by moving the assertions to the other part of the discussion section.

**Response**:	We have now stated the conclusion more simply in the Discussion section:

- *“In summary, we found no associations between SNPs related to OA presence and knee OA progression. However, 19 SNPs were newly associated with minJSW decrease and should be further validated to determine their role in knee OA. Given the rising incidence of OA and the lack of effective treatment options, continued efforts are needed to discover the basis of OA and OA progression, enabling patient stratification into more homogeneous subgroups.”.*

You can find these changes in lines 260 – 263.

2. Inclusion criteria for target patients
Patients with OA according to the ACR criteria were included, but with widely varying disease status, ranging from early OA with no clear objective findings to advanced OA (KL grade 4). Although there may be limitations due to the small number of patients included in the study, we suggest that an analysis limited to patients with KL grade 2 or higher (or grade 1 or higher) should be considered.

**Response**:	Due to the limited number of patients in this study, we have decided not to do a sub-group analysis. This is a limitation in our study, and therefore, we have extended our limitation section in the Discussion. We discuss the limitations regarding the wide variation of the disease status of the study population in the revised manuscript as follows:

- “*Known risk factors for OA, such as age, obesity, and sex, are a source of heterogeneity that complicates genetic research in OA. Genetic factors might even affect other prognostic factors (effect modification), further complicating analysis [2]. Our study population included patients at different stages of OA, varying from early to more advanced OA (KL grade 4). While we adjusted for several confounding factors, additional stratification of the population could be beneficial. However, this was not considered feasible due to the relatively small sample size*.”

You can find the changes in lines 234 – 238.

3. Definition of disease progression in knee OA
"Minimum joint space width" and "KOOS pain" have been used. Are these commonly used in past papers? I believe that these definitions are central to this study and should be stated in such a way that it is clear that the authors' definitions are appropriate. In particular, regarding "minimum joint space width," it should be clearly stated in the text how lateral joint space/medical joint space/PF joint space, etc. were treated. Although references are provided, I believe that it would be appropriate to include in the text the method used to take the images and the measurement sites used for the measurements.

**Response**:	The outcome measured “Minimum joint space width” (irregardless of medial/lateral) and “KOOS pain” are often used in previous research investigating progression of knee OA. We have now extended our Methods section by explaining the outcome measure in more detail. We have explained how the radiographs were obtained and how the scores were measured:

- “*Posterior-anterior weight-bearing semiflexed knee radiographs of the index knee were obtained to determine the minJSW using Knee Images Digital Analysis software and to grade the Kellgren Lawrence (KL) score [14, 16, 17]. The radiographs were acquired according to the protocol described by Buckland-Wright et al. at baseline and 24 months [18]. Pain was evaluated using the KOOS questionnaire (on a 0-100 scale, with a higher score indicating worse outcomes) [13]. More study details and baseline characteristics have been described previously [12, 19].*”

You can find these changes in lines 86 – 91.

The outcome definitions were previously decided in the IMI-APPROACH cohort. In line with this research, we have used the same OA progression outcomes. We have clarified this and explained why we deemed these outcome measures appropriate for our analysis. You can find these changes in lines 92 – 98.

4. Discussion
The results of this study are simple but important, and we believe that they should be thoroughly reviewed in light of previous findings. Are there no reports prior to this study regarding the two major results of "SNPs related to knee OA progression" and "Relationship and comparison between presence and progression of knee OA (including genetic and other factors) "? Please discuss the results of this study after citing the key reports, as I believe there are some, at least with respect to the latter.

**Response**:	We are not aware of any studies specifically looking at the association between SNPs and the progression of knee OA. There are known risk factors for OA, like sex and age, which are related to both knee-OA presence and progression (see, e.g. [1] ). Contrary to this, we seem not to see this for knee-OA related SNPs. We have now included a more elaborate discussion of previous research in the Introduction Section as follows:

- “*Many genetics studies on OA focus on the risk of OA in general or in specific joints rather than progressive outcomes [6, 7, 28, 29]. However, finding genetic variants associated with the risk of progression of OA will make it possible to define endotypes/phenotypic subtypes of OA. Some clinical risk factors for developing knee OA are also relevant risk factors for disease progression [30]. However, it is unknown whether genetic risk loci for presence of OA or knee OA are also relevant for progression of knee OA.*”

You can find the changes in lines 58 – 62.

[1] Sarmanova A, Fernandes GS, Richardson H, Valdes AM, Walsh DA, Zhang W, Doherty M. Contribution of central and peripheral risk factors to prevalence, incidence and progression of knee pain: a community-based cohort study. Osteoarthritis and Cartilage. 2018 Nov 1;26(11):1461-73. Doi: 10.1016/j.joca.2018.07.013

Specific points:

Background information on the subject patients
Very limited information is provided. Please consider adding information such as past history of knee trauma (post traumatic OA) and KOOS score other than pain.

**Response**:	We have added information on the subject participants of the study in the Results Section:

- *” The participants in IMI-APPROACH were mostly women (77,4%) and 66.5*±*7.1 years old on average (Table 1). The majority of participants (55%) had definite radiographic OA (KL score ≥ 2). The mean minJSW was 2.52*±*1.3 mm at baseline and 2.41*±*1.2 mm at 24 months; the mean decrease in minJSW over 2 years was 0.63*±*0.69 mm. Four patients did not agree to genotyping, and five patient samples did not pass quality control. After data preprocessing, 1.263.798 SNPs and 273 samples (62 males, 211 females) were available for analysis.”*

You can find the changes in lines 159 – 163.

Other information, such as KOOS score other than pain, is not used in this study. Therefore, we decided not to report this, but refer to the paper describing the characteristics of the IMI-APPROACH population. See lines 90 – 91: “*More study details and baseline characteristics have been described previously [12, 19].”.*

30 SNPs
How were these extracted? Were there any selection criteria?
Or were they arbitrarily selected by the authors from previous literature?

**Response**:	The SNPs were selected from previous literature, specifically two recent papers on genetics in OA [1,2]. From all SNPs mentioned in these papers to be related to OA in general or knee OA specifically, we had 30 SNPs that were also measured in our cohort. These SNPs were used to calculate our polygenic risk score. We have clarified the text regarding the extraction and selection of the SNPs in line with the above text:

- *“Previous Genome-wide association studies (GWAS) in large cohorts discovered in over 100 OA risk SNPs [7,9]. We selected all SNPs from these GWAS studies that were significantly associated (P < 5 × 10−8) with the presence of OA irrespective of the joint affected or knee OA and that were also present in the genotyping dataset of the IMI-APPROACH study (from now on referred to as 'OA presence SNPs’). This set of 30 OA presence SNPs was investigated in our cohort for an association with knee OA progression (see Table 2).”*

You can find the changes in lines 118 – 122.

[1]	Boer CG, Hatzikotoulas K, Southam L, Stefánsdóttir L, Zhang Y, Coutinho de Almeida R, et al. Deciphering osteoarthritis genetics across 826,690 individuals from 9 populations. Cell. 2021;184(18):4784-4818.e17. doi: 10.1016/j.cell.2021.07.038.

[2]	Aubourg G, Rice SJ, Bruce-Wootton P, Loughlin J. Genetics of osteoarthritis. Osteoarthritis Cartilage. 2022;30(5):636-49. doi: 10.1016/j.joca.2021.03.002.

Limitation
Please add "limitation" to the "discussion section".

**Response**:	We have elaborated on the limitations in our Discussion section. We have now addressed aspects such as the sample size, limitations of our outcome measures, and confounding factors. You can find the changes in lines 234 – 243.

**Responses to questions of reviewer #2**

Reviewer #2: Thank you for an important study in a field which deserves attention. Although the framework of the study seems to be of a good quality, the manuscript is a little unpolished and confusing. In general, the M&M and results sections could use more structure.

As far as I can tell, a full GWAS on the progression of OA has been performed, with several outcomes available, both radiological change (minJSW) and PROMS data (KOOS). The authors wanted to see if previous knee OA associated SNPs would be replicated, and to see if these 30 SNPs could be used as a PRS for knee OA progression. They have also done several functional analyses based on the results.

**Response**:	Thank you for your valuable and extensive feedback. Below, we respond to your specific remarks and suggestions.

Major comments:
1. The manuscript is confusing in its current form. I would present the data as a knee OA progression study with the primary and secondary outcomes from the current dataset in a more structured way, leaving the previous knee OA SNPs as a later, separate paragraph along with the PRS. As of now, it is difficult to understand the difference between the 30 known SNPs and the completely separate 19 SNPs from the current GWAS that was actually significant. I suggest switching Table 2 (previous knee OA SNPs) with Table S1 (Current associated SNPs). I would also use fig S1 A and B (Manhattan and QQ-plot for minJSW change) as main figures in the text. Some of the functional data as well as figures could also be included in the main body of the paper.

**Response**:	We have included the findings that were presented in Figs S1A and S1B and Table S1 in the main text, instead of the supplementary. We have more clearly separated the initial (exploratory) GWAS analysis aimed at evaluating the 30 SNPs previously identified as related to knee OA presence to knee OA presence. The PRS was based on these 30 ‘known’ SNPs’, as we have now explained more clearly.
You can find the changes regarding these methods in lines 106 – 128 and 148 – 155.

In response to your suggestion, we have also presented more detailed descriptions of methods and results in the main text that were previously in the Supplementary: we have moved Table S1 and Figures S1A and S1B to the main text. Furthermore, we have moved the ‘Functional analysis’ Section from the Supplementary to the main text.

The methods and results of the functional analysis that are described in more detail in the revised manuscript can be found in lines 136 – 146, and lines 187 – 202.

2. I would like there to be more details on the minJSW measurements, the SD is quite high compared to the average change between 0 and 24 months. A figure such as a box plot would be nice, as well as some discussion around the limitations of the measurement, precision etc.

**Response**:	We have discussed the minJSW measurements in more detail in the Methods Section (see also response to reviewer 1 point 3):

- “*Posterior-anterior weight-bearing semiflexed knee radiographs of the index knee were obtained to determine the minJSW using Knee Images Digital Analysis software and to grade the Kellgren Lawrence (KL) score [14, 16, 17]. The radiographs were acquired according to the protocol described by Buckland-Wright et al. at baseline and 24 months [18]. Pain was evaluated using the KOOS questionnaire (on a 0-100 scale, with a higher score indicating worse outcomes) [13]. More study details and baseline characteristics have been described previously [12, 19].*”

You can find these changes in lines 86 – 91.

To further clarify the SD of the minJSW, we have added the following to the results section:

- *” The participants in IMI-APPROACH were mostly women (77,4%) and 66.5*±*7.1 years old on average (Table 1). The majority of participants (55%) had definite radiographic OA (KL score ≥ 2). The mean minJSW was 2.52*±*1.3 mm at baseline and 2.41*±*1.2 mm at 24 months; the mean decrease in minJSW over 2 years was 0.63*±*0.69 mm.”*

You can find the changes in lines 159 – 161.

Furthermore, we have discussed the limitations of our outcome measures in more depth (see also point 4 of reviewer 1). We have now addressed aspects such as the sample size, limitations of our outcome measures, and confounding factors. You can find the changes in lines 230 – 243.

3. Technically, as several outcomes have been tested, the threshold for statistical significance should reflect this. With 6 outcomes, it should be (5x10-8)/6 if using Bonferroni correction.

**Response**:	The most commonly used threshold in GWAS analysis to determine the significance of associations between SNPs and a certain trait is a P-value of 5x10-8. This threshold is already very stringent regarding correction for multiple testing (the value is a Bonferroni corrected p-value of 0.05 that is based on the estimated number of SNPs that are tested within a GWAS analysis). Our primary outcome is the change in minJSW over time. Although we evaluated additional outcomes, we did not apply multiple testing corrections to these, as they are supplementary to the main results.
To further address your concern: if we were to conservatively apply a Bonferroni correction for all outcomes, 9 of the 19 SNPs we found would still remain significantly (p < 8.33x10^-9^) associated with minJSW change. Given our small sample size, the P-value of 5x10-8 is already very conservative and likely results in some missed significant associations. For this reason, we decided not to include results adjusted for multiple testing in the manuscript.

4. As the study is borderline in terms of power with regards to some of the outcomes (as apparent in several QQ-plots), the manuscript could do with elaboration on the general limitations of the study, PROMS data etc.

**Response**:	We have extended our discussion with a specific paragraph on the limitations of the study. This includes the limitations of PROMS data and the size of the study population. You can find the changes in lines 234 – 243.

Minor comments:
1. Some superfluous use of parentheses in the text, like (knee) OA.

**Response**:	We have made the use of these parentheses consistent throughout the text and reduced the unnecessary use of parentheses.

2. Write a little bit more about the functional analyses other than "incorporating various data sources" (p 8, line 160-161)

**Response**:	In response to your suggestion, we have discussed the functional analysis in more detail in the main text, instead of only in the Supplementary. Specifically, we have provided details on the data sources and the analytical approaches we have used for the functional analysis.

- The methods of the functional analysis are now described in lines 136 – 146.
- The results of the functional analysis are described in lines 186 – 202.

**Responses to questions of reviewer #3**

Reviewer #3: In this manuscript, the authors tested 30 published SNPs and found no association with knee osteoarthritis progression. This paper has several major issues, my comments are as below:
1. The authors only investigated 30 previously reported SNPs for OA progression, which is obviously too few and unredeemable. Ideally, the authors should generate their own genome-wide SNPs array data and test it again in IMI-APPROACH cohort.

**Response**:	Thank you for your feedback. The aim of our study was to examine whether known genetic markers for the risk of *presence* of knee OA could also play a role in the *progression* of knee OA. Our genotyping data was generated using the Illumina Omni2.5Exome-8 array v1.5 kit, which resulted in a genome-wide SNP array dataset.

The 30 SNPs were selected from previous literature, specifically two recent papers on genetics in OA [1,2]. From all SNPs mentioned in these papers to be related to OA in general or knee OA specifically, we had 30 SNPs that were also measured in our cohort. We have clarified the text regarding the extraction and selection of the SNPs in the methods section of the revised manuscript as follows:

- “*Previous Genome-wide association studies (GWAS) in large cohorts discovered in over 100 OA risk SNPs [7,9]. We selected all SNPs from these GWAS studies that were significantly associated (P < 5 × 10−8) with the presence of OA irrespective of the joint affected or knee OA and that were also present in the genotyping dataset of the IMI-APPROACH study (from now on referred to as 'OA presence SNPs’). This set of 30 OA presence SNPs was investigated in our cohort for an association with knee OA progression (see Table 2).”*

You can find these changes in lines 118 – 122 (See also our response to ‘Specific points’ of reviewer 1).

[1]	Boer CG, Hatzikotoulas K, Southam L, Stefánsdóttir L, Zhang Y, Coutinho de Almeida R, et al. Deciphering osteoarthritis genetics across 826,690 individuals from 9 populations. Cell. 2021;184(18):4784-4818.e17. doi: 10.1016/j.cell.2021.07.038.

[2]	Aubourg G, Rice SJ, Bruce-Wootton P, Loughlin J. Genetics of osteoarthritis. Osteoarthritis Cartilage. 2022;30(5):636-49. doi: 10.1016/j.joca.2021.03.002.

2. Each section of this paper is too brief and lacks sufficient depth. To enhance the quality and rigor of the paper, more content should be added to each section. For example, the introduction section could be expanded by including a more detailed background on the research topic, providing context to the field.

**Response**:	We have expanded the Introduction section. We discuss previous findings on known genetic loci for OA, the link between OA presence and OA progression, and the relevance of investigating the link between OA presence and OA progression risk factors. You can find changes regarding these points in lines 52 – 57, and 58 – 62 of the revised manuscript.

Furthermore, we have discussed the outcome measures we have used in more detail in the Methods section: lines 92 – 98 (see also our response to reviewer 2 point 2 and reviewer 1 point 3).

The functional analyses that were discussed in the Supplementary, have been moved to the main text. We have provided details on the data sources and the analytical approaches we have used for the functional analysis. The methods of the functional analysis are now described in lines 135 – 146. The results of the functional analysis are described in lines 186 – 202.

We have added a limitation section to the discussion. The limitations discussed are the size of the study population, the limitations of our outcome measures (such as the limitations of using PROMS), and confounding factors. You can find the changes in lines 234 – 243.

3. The SNP location was based on GRCh Build 37; however, the more advanced version, GRCh Build 38, has been released in 2013, which was more than ten years ago. The authors should definitely utilize the latest genome build and try to re-run the analysis.

**Response**:	The IMI-APPROACH project started about a decade ago. At that time, it was decided to use the GRCh Build 37 to perform the sequencing analysis. Our current dataset is, therefore, aligned with the GRCh Build 37. Recollecting the data using GRCh38 is not feasible. We do believe that our analyses remain valid and relevant, as GRCh37 continues to be a reliable reference for genomic studies.

**Responses to questions of reviewer #4**

Reviewer #4: In this study, the authors assessed whether a polygenic risk score (PRS) based on 30 genetic variants that have been associated with risk of knee osteoarthritis (OA) could be used to predict progression of knee OA. The authors found no association of the PRS to the progression of knee OA, but detected 19 significant genetic variants associated with a decrease of minimum joint space width over a 24 month period, an indication of worsening OA.

It is not surprising that the PRS is not significantly associated with knee OA progression given that none of the 30 PRS variants reached statistical significance in the GWAS. However, I have concerns about how these 30 SNPs were selected, please see my comments below.

The focus could be shifted away from assessing the polygenic risk score which was designed to predict the presence of OA, rather than the progression of OA, although this can still be included in the study as it is still valuable knowledge that variants for risk of knee OA do not overlap with those associated with progression.

Overall, there is enough data here for this study to be an independent examination of genetic markers (independent of the 30 known risk variants) associated with kneeOA progression. It could be further improved with more thorough examination of the 19 significant SNPs, and depending on data availability, the addition of transcriptomic analyses of changes over time or within-population differences that contribute to worsening OA. With some major revisions, I would recommend this study for publication.

**Response**:	Thank you for your valuable feedback and useful suggestions. Below, we respond to your specific remarks.

Please see my specific comments below:

Abstract:
- KOOS not defined
- Please italicise gene names on line 37 and throughout the manuscript.

**Response**:	We have now defined KOOS in the abstract and we changed all gene names throughout the manuscript to italics.

Methods:
- Was BMI recorded at 24 months? Is the change in BMI or high baseline BMI associated with worsened OA progression?

**Response**:	We have BMI records available at 24 months. We did not previously check whether a change in BMI or high baseline BMI was associated with worsened progression. We calculated the Pearson correlation coefficient between baseline BMI and change in minJSW (over 24 months), and between change in BMI (over 24 months) and change in minJSW. Both baseline BMI and change in BMI show very low correlation with minJSW change and were not statistically significant: r2 = -0.112, p-value = 0.958, and 0.046, p-value = 0.762, respectively. Therefore, we decided not to present results (further) corrected for BMI in our analyses in the revised manuscript.

- References in table 2 are not in reference list and does not follow same citation style. Please state if P values and OR from Table 2 are from the present study or from the cited source. It is also unclear as to how these 30 OA presence SNPs were selected. Presuming that Boer et al., 2021 (reference 6) is currently the largest GWAS study for knee OA, why were their 31 significant kneeOA SNPs not used for assessment and PRS in the current study?

**Response**:	We have changed the references in Table 2 to the proper citation style and we stated more clearly that the OR and p-values are derived from the cited studies:

- “*The PRS was calculated in PLINK v1.9 [21] using the effect size of the SNPs (as previously reported, see Table 2) multiplied by their presence and averaged over the number of non-missing SNPs in individual patients*.”

You can find the changes in lines 151 – 153.

The aim of our study was to examine whether known genetic markers for the risk of *presence* of OA or knee OA could also play a role in the *progression* of knee OA. The 30 SNPs were selected from previous literature, specifically two recent papers on genetics in OA [1,2]. From all SNPs mentioned in these papers to be related to OA in general or knee OA specifically, we had 30 SNPs that were also measured in our cohort. We have clarified the text regarding the extraction and selection of the SNPs in the methods section of the revised manuscript as follows:

- *“Previous Genome-wide association studies (GWAS) in large cohorts discovered in over 100 OA risk SNPs [7,9]. We selected all SNPs from these GWAS studies that were significantly associated (P < 5 × 10−8) with the presence of OA irrespective of the joint affected or knee OA and that were also present in the genotyping dataset of the IMI-APPROACH study (from now on referred to as 'OA presence SNPs’). This set of 30 OA presence SNPs was investigated in our cohort for an association with knee OA progression (see Table 2).”*

You can find the changes in lines 118 – 122. (See also our response to ‘Specific points’ of reviewer 1 and reviewer 3 point 1).

[1]	Boer CG, Hatzikotoulas K, Southam L, Stefánsdóttir L, Zhang Y, Coutinho de Almeida R, et al. Deciphering osteoarthritis genetics across 826,690 individuals from 9 populations. Cell. 2021;184(18):4784-4818.e17. doi: 10.1016/j.cell.2021.07.038.

[2]	Aubourg G, Rice SJ, Bruce-Wootton P, Loughlin J. Genetics of osteoarthritis. Osteoarthritis Cartilage. 2022;30(5):636-49. doi: 10.1016/j.joca.2021.03.002.

Additionally, SNPs rs2605100, rs10831475 and rs9981884 were listed under the TJR (total knee and/or hip replacement) phenotype, while rs13107325, rs216175 and rs10405617 were listed under AllOA phenotype instead of kneeOA in Boer et al., 2021.

**Response**:	The aim of our study was to examine whether known genetic markers for the risk of OA in general (irrespective of specific joint) or knee OA specifically could play a role in the progression of knee OA. SNPs rs2605100, rs10831475, and rs9981884 were indeed listed under TJR, but were also known to associate with knee OA. The rs13107325, rs216175, and rs10405617 SNPs were included as these were also related to OA in general. We have clarified how we selected the SNPs in our methods section. See also our response to your previous comment.

- There were 297 patients in the cohort (Line 70), however blood was only collected from 288 patients (Line 90); why were 9 samples excluded?

**Response**:	Four samples were excluded from the analysis because these patients chose not to agree to genotyping. Another five samples did not pass quality control before genotyping (contaminated samples) and were excluded. We have clarified in our Methods section how many samples were excluded after each quality control steps. You can find the changes in lines 110 – 117.

In the Results section, the number of patients that were included in our analysis is also specified

- *“Four patients did not agree to genotyping, and five patient samples did not pass quality control. After data preprocessing, 1.263.798 SNPs and 273 samples (62 males, 211 females) were available for analysis.”*

You can find the changes in lines 161 – 163.

- How was relatedness assessed? Please provide kinship values and cutoffs used.

**Response**:	We have added details on the relatedness in the Methods section as follows:

- *“Patient relationships more closely related than second cousins (proportion of IBD sharing > 0.03125) were identified and one from each pair was removed (n = 4).* “

You can find the changes in lines 111 - 113.

- Further details are needed for the ancestry assessment. How many reference samples were used to compare against the cohort, and were they derived from publicly available data? If so, please cite the source. How many variants were used for the ancestry assessment? It would be good to show a PCA plot of the samples in the supplement, including those from the reference ancestries.

**Response**:	We have added the information regarding the ancestry assessment in the Methods section as follows:

- *“Patient relationships more closely related than second cousins (proportion of IBD sharing > 0.03125) were identified and one from each pair was removed (n = 4). The same set of LD-pruned SNPs was used in the assessment of the patients’ ancestry. Principal component analysis of these SNPs against three sample populations from the 1000 Genome project (60 samples with European ancestry (CEU), 60 samples with African ancestry (Yoruba, YRI), 90 samples with East Asian ancestry (Japanese/Chinese, JPT)) were used to exclude samples of non-European ancestry (n = 6, see Fig S1) [22]. “*

You can find the changes in lines 111 - 116.

In addition, we included a PCA plot of the samples against the reference samples in the supplementary, see Figure S1.

- Was multiple testing correction done for the regression analyses?

**Response**:	The most commonly used threshold in GWAS analysis to determine the significance of associations between SNPs and a certain trait is a P-value of 5x10-8. This threshold is already very stringent regarding correction for multiple testing (the value is a Bonferroni corrected p-value of 0.05 that is based on the estimated number of SNPs that are tested within a GWAS analysis). Our primary outcome is the change in minJSW over time. Although we evaluated additional outcomes, we did not apply multiple testing corrections to these, as they are supplementary to the main results. See also our response to point 3 reviewer 2.

Results:
- Considering that none of the 30 OA presence SNPs were associated with progression outcomes, it is not surprising that the PRS was not significantly associated with progression either given it is a small set of SNPs. It would be interesting for the authors to examine a PRS based on the 19 significant SNPs from the minJSW decrease GWAS. Is it possible to predict whether a patient will have decreased minJSW based on these 19 SNPs?

**Response**:	As an additional analysis, we have created a second PRS based on the 19 new SNPs that were associated with the minJSW decrease. The results are reported as follows:

- *“We performed an exploratory analysis using the 19 SNPs newly associated with the minJSW decrease to create a second PRS (-0.040 ± 0.053, mean ± sd). Linear regression analysis, corrected for age, sex, disease duration, and baseline minJSW, revealed a significant association of this PRS with the minJSW decrease (β = 7.61, 95% CI [6.07, 9.15], p = 6.899e-19, adjusted R2 = 0. 26). “*

You can find the change in lines 209 – 212.

- How many base pairs away were considered a ‘nearest gene’ for Table S1? There are several genes in the table with “Na” for nearest gene, how far away is the closest gene?

**Response**:	The nearest genes of a SNPs within 2kb upstream of the 5’ end of a gene transcript or within 2kb downstream of the 3’ end of a transcript are reported. We have added this information to Table 3 (was previously Table S1).

- *“The nearest protein coding gene to the SNP (within 2kb upstream of the 5’ end of a gene transcript or within 2kb downstream of the 3’ end of a transcript) is named.”*

You can find the changes in lines 179 – 181.

Please state the odds ratio with 95% confidence intervals or beta coefficient with standard error for each SNP as well. It may be worth examining LD around all the top hits and investigating what other genes are nearby. P < 5 × 10−8 is quite a strong association considering the sample size so the other associations shouldn’t be discounted.

**Response**:	We have added the beta coefficient and standard error for the 19 significant SNPs in Table 3. You can find the changes in lines 177 – 179.

We investigated all 19 significant SNPs and examined whether they were in linkage disequilibrium with other significant SNPs. We found two loci: three SNPs in LD (r2 > 0.8) located near *PLCL2* (chromosome 3) and six SNPs in LD located near *CDYL2* (chromosome 16). The remaining 10 significant SNPs were in or near *NTNG1*, and the non-coding genes LINC02619, LINC02379, and ARLNC1. These 10 SNPs were not in LD with other significant SNPs and, therefore, were not further explored.

- As supplementary S1 and S2 forms a major component of this study, these sections belong in the main text.

**Response**:	We have included the findings that were presented in Figs S1A and S1B and Table S1 in the main text, instead of the supplementary. We have discussed the functional analysis in the main text instead of the Supplementary. Specifically, we provided details on the data sources and the analytical approaches we have used for the functional analysis.

- The methods of the functional analysis are now described in lines 136 – 146.
- The results of the functional analysis are described in lines 186 – 202.

See also our response to reviewer 2 point 1.

Regarding the available RNA-sequencing data, when were these samples collected (baseline, 12 months, 24 months)? If there is access to RNA-seq data for all three time points, of even for two time points, it would be valuable to assess the transcriptomic changes with worsening knee OA. If there is only one sampling for RNA-seq, it would be valuable to compare transcriptional differences within the population, for patients with the greatest progression compared to those with the least.

**Response**:	We only have RNA-sequencing data at baseline, with no data available for the 12- or 24-month time points. This has now been described in the text as follows:

- *“The effect of SNPs in or near genes on the gene expression levels was evaluated using baseline RNA-sequencing data from IMI-APPROACH (see Supplementary Information S1) [19]”*

You can find the changes in lines 138 – 140.

Due to the limited number of patients in this study, we have decided not to divide the population into subgroups for further analysis. The primary aim of our study was to examine whether known genetic markers for the risk of *presence* of knee OA could also play a role in the *progression* of knee OA. Analyzing RNA-sequencing data in relation to disease progression in sub populations would extend beyond the intended scope of this study.

- Please state an r^2 value in which “strong” LD is defined, or report the range of r^2 values for the SNPs that are in strong LD.

**Response**:	We should indeed specify the r^2^ value, which we have now reported in the text. You can find the change in line 189.

Discussion:
- Citation for line 163 lacking (“..which is implicated in the regulation of genome stability as well as mitosis…”)

**Response**:	We have added a citation. You can find the change in line 224-225.

- I could not access <https://github.com/MiekeBvz/IMI-APPROACH_Genomics.git>

**Response**:	We have made the link accessible.

- Any discussion points for PLCL2? What is the function of this gene?

**Response**:	We have extended our Discussion section. A discussion on PLCL2 is now part of the discussion in the revised manuscript as follows:

- *“We did not observe a significant association between the expression of PLCL2 and minJSW decrease. However, it was previously found that PLCL2 is downregulated in knee OA patients with damaged cartilage compared to those with undamaged cartilage [39]. PLCL2 should be further investigated to determine its role in the progression of knee OA.”*

You can find the changes in lines 227 – 229.

- As above, the other significant hits should not be ignored, I think some discussion is warranted around them, including those near or in lncRNAs since ncRNAs have shown a role in progression of OA (e.g. Ji et al., 2023, Am J Hum Genet 110(4):606-624, PMID: 36868238).

**Response**:	We have included the findings that were presented in Figs S1A and S1B and Table S1 in the main text instead of the supplementary. We have also discussed the implications of the significant results more elaborately, highlighting what implications the SNPs in the protein-coding genes, as well as the SNPs in the lncRNAs, could have. You can find the changes in lines 220 – 233.

Supplementary:
- Figure S1 – not sure why there is chromosome 0 in the Manhattan plots – are they possibly standards used by Illumina? Also please rename chromosomes 23-26 as X, Y, and MT or note it in the figure legend. The axes should also be adjusted for figures C-L, the y-axis limit could be set to 10. In the figure legends, please state for each GWAS if it was a linear or logistic regression.

**Response**:	We have clarified what the numbers of the chromosomes in the Manhattan plots mean in the figure legends. We have also specified for each GWAS if it was linear or logistic regression in the figure legends.

You can find the changes in lines 9 – 24 of the Supplementary.

- Figure S5 – please state the units for change in minJSW.

**Response**:	We have added the unit for change in minJSW in Figure S5.

**Responses to questions of reviewer #5**

Reviewer #5: In this study, Bentvelzen et al. asked if previously identified genetic markers of knee Osteoarthritis (OA) presence are also risk makers of knee OA progression. Authors accessed a cohort of 297 patients from the IMI-APPROACH prospective clinical study, and used the study progression outcomes to test for genome-wide associations with markers present in the Illumina Omni2.5Exome-8 array v1.5 kit. The study details and clinical characteristics are remitted to references 2 and 9 in the manuscript (Deveza et al, 2019; van Helvoort et al, 2021)*. The primary progression outcome was decrease in minimum joint space width (minJSW) in mm over 24 months. The researchers genotyped 288 of the patients, and 273 of these samples (62 male, 211 female, all of European ancestry) along with 1.263.798 SNPs with minor allele frequency > 5% passed GWAS quality control parameters and were included in the analysis. The authors identified, within the genomic array kit, 30 variants as previously associated with OA presence and investigated for their association with OA progression outcomes in their cohort. They used linear regression for the continuous OA progression outcomes and logistic regression for binary progression outcomes with the PLINK v1.9 software. Calculations were controlled for “sex, age, time since OA diagnosis, and baseline minJSW or KOOS pain depending on the outcome”. The authors have also calculated a weighted polygenic risk score (PRS) on the 30 selected variants and tested for its association with the same outcomes using the same software and controlling for the same covariates. The results showed no evidence of association between the variants (which had been previously associated with OA presence) with the disease progression, and likewise, the PRS of these variants was not associated with progression. The lack of association suggested that different outcomes (i.e., OA presence vs. OA progression) could have different genetic backgrounds. Indeed, additional analysis revealed that 19 other SNPs, which had not been previously associated with knee OA presence, were associated with the knee OA progression outcome: minJSW decrease. Upon bioinformatic mining of the associated SNPs and interrogation of another dataset from the same cohort - IMI-APPROACH cohort gene expression - the authors selected two genes (genes PLCL2 and CDYL2) each located in the vicinity of clusters of associated variants, that were in linkage disequilibrium, for further exploration. There was no association between the OA progression outcomes with gene expression (non-normalized counts) for PLCL2, but a small trend was observed for the gene CDYL2. Interestingly, upon exploration of the GTEx eQTL database, 6 of the variants located around gene CDYL2 significantly associated with OA progression were also associated with lower expression of the gene in the muscle skeletal tissue.
The study warrants publication, not only due to the significance of reporting “negative results”, but also because it underscores, as the authors suggest, the value of exploring a broad range of disease traits and (endo)phenotypes beyond primary disease classification. This approach could lead to uncovering of novel targetable pathways - currently hidden within more heterogeneous patient cohorts - potentially improving patient care. The authors found a secondary association with progression of OA, though they advise caution in interpretation due to the study’s sample size. I believe that with a few concerns addressed, confidence in the study’s findings could be strengthened.
Overall, my first point of criticism is that the analysis lacks sufficient detail in the description of datasets and methods and even on the choices made during the analysis. This lack of transparency limits clarity, interpretability and reproducibility. With a few adjustments and a more thorough discussion, this study could not only gain credibility but also potentially open up exciting new avenues for research. My major points of concern are the following:
1. Power calculations should be explicitly shared. The authors mention that the study is relatively small, but do not provide quantitative data to justify this claim. It is essential to include power calculations in the manuscript for the reader to assess the significance of the reported associations.

**Response**:	Thank you for your detailed and valuable feedback.

We recognize the importance of understanding the statistical power in assessing the significance of genetic associations. As we made use of an existing cohort for which the current analyses were not part of the primary analysis, no formal a priory sample size calculation was performed. Particularly for genome-wide association studies, larger sample sizes are typically used and required to detect genetic effects [1]. Therefore, we have acknowledged the potential limitations of our relatively small sample size in the Discussion section of the manuscript. However, we analyzed our primary progression outcome in the natural continuous scale (change in minJSW, rather than using an arbitrary cut-off for the presence of progression) to optimally and efficiently analyze our data. Furthermore, one could argue that the fact that we identify statistically significantly associated SNPs with progression, using a p-value appropriately correcting for multiple testing in GWAS studies [2] already with this small sample size, may indicate that there may also be other SNPs related to progression. We have now added this information to the part on sample size in the limitation section of the discussion of our revised manuscript.

You can find the changes in lines 226 and 248 – 254.

[1] Jiang W, Yu W. Power estimation and sample size determination for replication studies of genome-wide association studies. BMC genomics. 2016 Jan;17:19-32. doi: 10.1186/s12864-015-2296-4

[2] Johnson RC, Nelson GW, Troyer JL, Lautenberger JA, Kessing BD, Winkler CA, et al. Accounting for multiple comparisons in a genome-wide association study (GWAS). BMC genomics. 2010 Dec;11:1-6. doi: 10.1186/1471-2164-11-724

2. Sufficient control for confounding effects needs to be better addressed. In the discussion, the authors write: Lines 166-9: “Known risk factors contributing to the likelihood of OA, such as age, obesity, and sex (…). While we adjusted for these factors, further stratifying the population could be beneficial, but it was not feasible in our relatively small sample.” – however, in the methods section, authors don’t report to have adjusted for obesity: Lines 102-3: “(…) while adjusting for sex, age, time since OA diagnosis, and baseline minJSW or KOOS pain depending on the outcome.” This lapse could be a minor issue, but a closer look at the results got me intrigued on whether the associations reported could be confounded with traits which have been previously reported as associated with the genes highlighted as associated and which could likely be risk factors for OA progression. Namely, SNPs in the vicinity of the genes CDYL2 and ARLNC1 have been previously associated with traits such as “heel bone mineral density”, “waist-hip ratio”, “BMI-adjusted waist-hip ratio” and “body height” amongst others (as seen on a quick search on the GWAS catalog for both genes ARLNC1 and CDYL2, at <https://www.ebi.ac.uk/gwas/genes/ARLNC1> and <https://www.ebi.ac.uk/gwas/genes/CDYL2> ). Some of these variants could be in linkage disequilibrium with the variants associated with OA progression and even if not, they could still be confounded. The authors should describe which relevant clinical variables they have access to, rather than referencing other publications. They should adjust for all likely confounders present in the data, particularly those that are known or likely risk factors for OA and have previously been associated with the genes identified in this study. Additionally, the authors should account for the fact that some covariates may not be independent. Given concerns about power loss, they could prioritize confounders based on effect size, and if not adjusting for all confounders, at least include those with the largest effect sizes.

**Response**:	Confounding effects are critical to consider in analyses, especially for complex traits like OA or OA progression. In this paper, we have considered risk factors that can contribute to the likelihood of OA or OA progression as confounders in our GWAS analysis. Most risk factors that are currently recognized for the incidence of knee OA are not well established for the progression of knee OA. The evidence for an association between knee OA progression and BMI remains conflicting, as was found by previous reviewers [1-3]. Consequently, in our analyses, we have corrected for sex, age, and the baseline value of the phenotype of interest (e.g. minJSW or Kellgren and Lawrence score) but did not include BMI as a covariate.

To evaluate the specific concern regarding BMI as a potential confounder, we have calculated the Pearson correlation coefficient between BMI and change in minJSW. The correlation is very small and not statistically significant: r2 = -0.112, p-value = 0.958 (see also our response to reviewer 4 point 2). This result suggests that baseline BMI is not strongly associated with worsened progression (change in minJSW).

Furthermore, we have re-run our GWAS analysis in Plink, this time also correcting for baseline BMI along with sex, age, time since diagnosis and baseline minJSW. Correcting for BMI resulted in a few minor changes in our results. Six of the 19 SNPs significantly associated with minJSW decrease had a slightly reduced significance level after correction for BMI, falling just below the GWAS threshold of 5e-08. These SNPs included one SNP near *PLCL2* (rs6442653, p-value was 4,55E-08 and after correction p-value = 2,08E-07), while the other 3 SNPs near *PLCL2* remained significant and were in linkage disequilibrium with rs6442653. Furthermore, one SNP near LINC02619 (rs76660267) and one SNP near LINC02379 (rs71610153) were not significant after correcting for BMI (rs76660267 p-value was 4,30E-08 and became 1,28E-07; rs71610153 p-value was 2,72E-08 and became 7,00E-08). Lastly, three SNPs were not significant that are not near any coding or non-coding RNA (rs116117775 p-value was 2,35E-08 and became 5,42E-08; rs16902074 p-value was 4,19E-08 and became 9,37E-08; rs73153427 p-value was 2,66E-08 and became 2,63E-07).

Importantly, all SNPs near *CDYL2* and *ARLNC1*, previously associated with traits such as BMI, remained significant after correcting for BMI. This suggests that the observed associations in these regions are not driven by BMI-related confounding in our dataset.

Including BMI as a covariate does not result in substantial changes to the main conclusions of our study. Given the lack of significant association between BMI and minJS, and the relatively minimal impact of BMI adjustment on our GWAS results, we decided to keep the original results without BMI adjustment in the revised manuscript.

1. Bastick AN, Belo JN, Runhaar J, Bierma-Zeinstra SM. What are the prognostic factors for radiographic progression of knee osteoarthritis? A meta-analysis. Clinical Orthopaedics and Related Research®. 2015 Sep 1;473(9):2969-89.
2. Belo JN, Berger MY, Reijman M, Koes BW, Bierma‐Zeinstra SM. Prognostic factors of progression of osteoarthritis of the knee: a systematic review of observational studies. Arthritis Care & Research: Official Journal of the American College of Rheumatology. 2007 Feb 15;57(1):13-26.
3. Chapple CM, Nicholson H, Baxter GD, Abbott JH. Patient characteristics that predict progression of knee osteoarthritis: a systematic review of prognostic studies. Arthritis care & research. 2011 Aug;63(8):1115-25.

3. A more in-depth exploration of the gene PLCL2 and its previously associated traits may provide a basis for a functional hypothesis or reveal additional confounding effects. Notably, markers within or near the gene PLCL2 have been extensively linked with immune traits, including (auto-)inflammatory conditions such as Rheumatoid Arthritis (RA) and Psoriasis (<https://www.ebi.ac.uk/gwas/genes/PLCL2>.) It is also known that conditions such as Psoriatic Arthritis (PsA) are frequently undiagnosed and can be misdiagnosed for RA and OA (Saalfeld et al, 2021, <https://www.ncbi.nlm.nih.gov/pmc/articles/PMC8572231>). This warrants the inclusion of inflammatory markers (e.g., serologic) and disease history (e.g., psoriasis and other inflammatory diseases) as covariates in the GWAS analysis. After such adjustments, whether the association is confirmed or excluded as confounded by inflammatory disease covariates, the rigor of the results will be improved, and perhaps the authors will be more confident in advancing the study to a more comprehensive stage. For example, identifying an associated variant could facilitate earlier diagnosis of PsA in a patient of this study, allowing for earlier treatment and potentially reducing permanent joint damage. Alternatively, it could inform future studies aimed at developing diagnostic tools and impacting patient care.

**Response**:	Conditions such as psoriatic arthritis and other inflammatory diseases may overlap with OA. To account for this, secondary knee OA was an exclusion criterium for the IMI-APPROACH cohort (secondary knee OA due to, for example, severe leg deformity, inflammatory joint disease, severe chondrocalcinosis, Paget’s disease of the bone, ochronosis, acromegaly, haemochromatosis, Wilson’s disease, osteochondritis dissecans, haemophilia) [1].

In addition, we had access to the inflammatory marker hsCRP: high-sensitive C-reactive protein blood levels. We have calculated the Pearson correlation coefficient between hsCRP and change in minJSW to check whether an association exists. The correlation is very small and not statistically significant: r2 = -0.046, p-value = 0.764.

As with our answer to your previous question, we have re-run our GWAS analysis in Plink this time correcting for baseline hsCRP, as well as for the combination of hsCRP and BMI. Correcting for hsCRP did not result in any changes in the significant SNPs. When correcting for hsCRP and BMI, we found similar results as when we only corrected for BMI (i.e. six SNPs were not significant after correcting for BMI).

The hsCRP does not seem to be a confounder in our patient dataset. Therefore, we decided not to present results (further) corrected for hsCRP in our analyses in the revised manuscript.

We have added an additional discussion point to address further the need to correct for confounders. You can find the changes in lines 234 – 238.

[1] van Helvoort EM, van Spil WE, Jansen MP, Welsing PMJ, Kloppenburg M, Loef M, et al. Cohort profile: The Applied Public-Private Research enabling OsteoArthritis Clinical Headway (IMI-APPROACH) study: a 2-year, European, cohort study to describe, validate and predict phenotypes of osteoarthritis using clinical, imaging and biochemical markers. BMJ Open. 2020;10(7):e035101. doi: 10.1136/bmjopen-2019-035101.

4. The gene expression data must be normalized. Without adjusting for read depth, comparison across different libraries is not valid, rendering both the small association with CDYL2 expression and the lack of association with PLCL2 expression unreliable. Additionally, I could not find any explicit details in the manuscript regarding the type of gene expression experiment used (it appears to be RNA-Seq) and it is not mentioned in the methods section the kind of biological material used. These issues should be addressed and clarified.

**Response**:	We have provided details on the RNA-sequencing data in Supplementary Information S1.

Other minor opportunities for improvement, include:
1. I commend the authors to share the analysis steps of the study and analysis in a GitHub repository (Line 180). However, the GitHub repository either does not exist or is currently not publicly accessible. This issue should be addressed.

**Response**:	We have made the link accessible.

2. I recommend putting figures pertaining to results directly answering the main questions of the study in a more prominent position instead of supplementary data. I would also suggest including all the methods description in a single methods section.

**Response**:	Important findings that were presented in the Supplementary, are now included in the main text. We have included the findings that were presented in Figs S1A and S1B and Table S1 in the main text, instead of the Supplementary. We have also presented the methods and results of the ‘Functional analysis’ in the main text, which was previously in the Supplementary.

The methods and results that are now described in the main text of the revised manuscript can be found in lines 136 – 146, and lines 186 – 202. See also our response to point 1 of reviewer 2 and point 2 of reviewer 3.

3. As already stated, a) data parameters relevant to the study should be revealed; b) all methods for which there are results shown should be adequately and explicitly described.

**Response**:	We have now extended our Methods section by explaining the outcome measures in more detail. Among others, we have explained how the radiographs were obtained and how the scores, such as minJSW and Kellgren Lawrence, were measured:

- “*Posterior-anterior weight-bearing semiflexed knee radiographs of the index knee were obtained to determine the minJSW using Knee Images Digital Analysis software and to grade the Kellgren Lawrence (KL) score [14, 16, 17]. The radiographs were acquired according to the protocol described by Buckland-Wright et al. at baseline and 24 months [18]. Pain was evaluated using the KOOS questionnaire (on a 0-100 scale, with a higher score indicating worse outcomes) [13]. More study details and baseline characteristics have been described previously [12, 19].*”

You can find these changes in lines 86 – 91.

The outcome definitions were previously decided in the IMI-APPROACH cohort. In line with this research, we have used the same OA progression outcomes. We have clarified this and explained why we deemed these outcome measures appropriate for our analysis. You can find these changes in lines 92 – 98. See also our response to reviewer 1 point 3 and reviewer 2 point 2.

Furthermore, we added the methods and parameters used to assess ancestry and relatedness in the genetics data during data pre-processing. See also our response to reviewer 4 regarding ‘Discussion’. You can find these changes in lines 111 - 117.

We have presented the methods and results of the ‘Functional analysis’ in the main text, which was previously in the Supplementary. The methods and results of the functional analysis that are described in the main text of the revised manuscript can be found in lines 135 – 146, and lines 186 – 202.

4. General image readability should be improved (e.g., Figure S2 is not readable even after zooming in.)

**Response**:	We have generated new images with higher quality and better readability in the revised Manuscript and Supplementary.

5. Images and plots should be adequately labeled (e.g., in figure S3, the gene names could be annotated as title instead of only on the description of the figure. Overall visualization could be improved, maybe with violin plots instead of boxplots. Indeed, a good example of how to show this data is exemplified in Figure S4.)

**Response**:	We have improved the figure legends and labelling in the Manuscript and Supplementary.

6. A better description of the figures and tables should be attempted (e.g., in Table S1, explain what “Na” means – maybe by explaining the criteria used for “nearest gene”?)

**Response**:	Thank you for this feedback. The nearest genes of a SNPs within 2kb upstream of the 5’ end of a gene transcript or within 2kb downstream of the 3’ end of a transcript are reported. NA means that the SNP is not within 2kb upstream of the 5’ end of a gene transcript or within 2kb downstream of the 3’ end of a transcript. We have added this information to the table. You can find the changes in lines 179 – 181. Furthermore, we have improved the descriptions of the other figures and tables.

7. Authors should search for minor grammatical inconsistencies across the manuscript.

**Response**:	We have carefully reviewed the manuscript for grammatical inconsistencies and corrected any minor errors to improve readability and clarity.

* Perhaps the authors would like to confirm if both these references are correct.

**Response**:	The correct references are van Helvoort et al. 2020 and van Helvoort et al, 2021. We have extended our Methods section and provided more details on the measurement and clinical characteristics. See also our response to your minor comment 3.
