# Supplementary material for: Genetic markers for knee osteoarthritis presence are not associated with disease progression - data from the IMI-APPROACH cohort
Source: PLoS One. 2025 Jun 24;20(6):e0325819. doi: 10.1371/journal.pone.0325819 (PMC12186935; doi:10.1371/journal.pone.0325819)
Supplement: Attachment — Submitted filename: Response_to_Reviewers_auresp_2.docx [file pone.0325819.s013.docx]

**We thank the editor and reviewers for the helpful comments by which we feel the manuscript has improved. Please find an itemized response to all comments below (in blue). Hopefully, we answered all questions and addressed all comments to the intention and satisfaction of the reviewers.**

Journal Requirements:

Please review your reference list to ensure that it is complete and correct. If you have cited papers that have been retracted, please include the rationale for doing so in the manuscript text, or remove these references and replace them with relevant current references. Any changes to the reference list should be mentioned in the rebuttal letter that accompanies your revised manuscript. If you need to cite a retracted article, indicate the article’s retracted status in the References list and also include a citation and full reference for the retraction notice.

**Response**:	Thank you for your feedback. We have checked the Reference list and ensure that it is complete and correct. We have added one reference to the Reference list as part of a response to reviewer 2.

Additional Editor Comments :

The Editor reiterates his previous recommendation to report the manuscript strictly following the STROBE Checklist for cohort studies (<https://www.strobe-statement.org/>). In particular, the methods section should include all specific subsections suggested in the STROBE Checklist. Please include section titles as suggested by STROBE : 1) Study design; 2) Setting; 3) Participants; 4) Variables; etc., with slight adaptations if necessary. Please note that this will improve readability and clarity of the manuscript.

**Response**:	We have updated the manuscript to adhere to the STROBE Checklist.

Reviewer #1: All comments have been appropriately addressed, and I judge that it is acceptable to consider publication in this academic journal.

Reviewer #2: I thank the authors for their thorough review of this paper. It is now much more coherent and easier to read and follow along in the process. They have also addressed the concerns of myself and the other reviewers. However, in the revision process, they have added some PRS data that I would like to address as a major concern:
The topic of PRS and their usage and utility in clinical work is much debated. Still, a basic principle of PRS is to use a baseline dataset(s) to determine risk variants and their effect sizes and applying this to an independent target dataset(s) to see if the risk is transferable and quantifiable. In this paper, after not finding a knee OA presence PRS to be relevant to knee OA progression, the authors have used the 19 significant SNPs in their progression dataset as a PRS within their own dataset. This leads to an overfitting of the model, as seen with the exceptional p-value. Also, in pure scientific terms this approach makes little sense. Taking known risk variants for OA progression in a sample and testing if they are predictors of OA progression in the same sample will of course give positive results. Thus, I feel that this part of the paper should be removed. This concerns page 11-12 Line 209-212 and page 13 line 249-251.

**Response**:	Thank you for your feedback. We agree that the result of the additional PRS (calculated in the same sample) has limited interpretability. Partially based on a previous remark from another reviewer, we still think that including this finding is of benefit for the reader, to obtain a complete picture of the found results and verify that the PRS is indeed significant. We changed the sentences related to the additional PRS to further emphasize that these results are included for completeness and verification, not to interpret them as validated findings.

We have changed the description of the PRS in the results section as follows:

- “*For the sake of completeness, we performed an exploratory analysis using the 19 SNPs newly found to be associated with the minJSW decrease to create a second PRS (-0.040 ± 0.053, mean ± sd). Based on a linear regression analysis, corrected for age, sex, disease duration, and baseline minJSW, a significant association of this PRS with the minJSW decrease was found (β = 7.61, 95% CI [6.07, 9.15], p = 6.899e-19, adjusted R2 = 0. 26).”*

Furthermore, we have changed the description of the PRS in the results section as:

- “*To verify that a PRS based on these SNPs would also be significant, an additional PRS was created using these SNPs and indeed showed a significant association with minJSW decrease. These results were calculated in our dataset and not validated externally, so they should be interpreted with caution.*”

You can find these changes in lines 210 – 213 and 253 – 256 of the revised manuscript respectively.

Minor comments:
1) A few points in the abstract: Page 2 line 24-25: “Some genomic markers were previously associated with knee OA presence.” Please rephrase along the line of “ Several genomic markers have been previously associated with knee OA presence.”. The current sentence is a little unpolished.

**Response**:	We have changed the sentence as follows:

- “*Several genomic markers have been associated with knee OA presence*.”

You can find the changes in line 24 of the revised manuscript.

In the abstract and also later in the text I would advice to write something like "Kellgren Lawrence (KL) OA score" or in some other way specifying what the KL score is scoring.

**Response**:	We have changed the text about the Kellgren Lawrence score in the abstract to better clarify what the KL score is used for. We included changes as follows:

- “*Knee OA progression definitions were based on the decrease in minimum joint space width in mm (minJSW; primary outcome), increase in pain on the Knee injury and Osteoarthritis Outcome Score (KOOS), and presence of radiographic OA (based on the Kellgren Lawrence score) over 24 months.”*

You can find the changes in lines 28 – 30.

Furthermore, we state in the methods section in lines 101 – 102 the following: “radiographic OA (KL score ≥ 2 at 2 years)”. We feel that this clarifies what the KL score is used for.

Also in the abstract line 31-32: "We associated SNPs in a genome-wide association analysis..." is not very well formulated. A more correct phrase would be "We did a SNP based genome-wide association analysis.." or "We tested for association..".

**Response**:	We have changed the sentence as follows:

- “*We performed a SNP based genome-wide association analysis using the disease progression definitions*.”

You can find the changes in line 32 of the abstract.

Finally, in line 36, "Unexpectedly, nineteen new variants" is meant to separate the presence SNPs from the progression SNPs. In order to not confuse these variants with de novo etc, I would advice to say "nineteen different" or "separate" or something along those lines.

**Response**:	We have changed the sentence to better emphasize that the 19 SNPs are different from the *presence* SNPs:

- “*Unexpectedly, nineteen different variants were associated significantly (P<5×10−8) with minJSW decrease.*”

You can find the changes in lines 35 – 36.

2) In the main text, page 6 line 124, you also use the term "were associated with" when describing the testing itself. As above, please rephrase.

**Response**:	We have adjusted the text as follows:

- *“We performed a genome-wide association analysis using the OA progression outcomes. We included the 30 OA presence SNPs, as well as all other SNPs measured in the study, in the analysis (to include other SNPs that may be in linkage disequilibrium with or in the same haplotype block as one of the 30 OA presence SNPs). The analysis was performed using linear regression in PLINK 1.9 [21], while adjusting for sex, age, time since OA diagnosis, and baseline minJSW or KOOS pain depending on the outcome.”*

You can find the changes in lines 124 – 128 of the revised manuscript.

3) Page 11 line 188-189, please take the text in the parenthesis out as a separate sentence.

**Response**:	We have removed the parenthesis, and we have rewritten the sentences as follows:

- *“Three SNPs on chromosome 3 are in strong linkage disequilibrium: r2 > 0.8 (Fig S3A). LD describes the extent to which one SNP is inherited with or associated with another SNP within the population.”*

You can find the changes in lines 189 – 190.

4) Page 11 line 195-197: You state that CDYL2 is associated with lower gene expression in skeletal muscle tissues. Although very speculative, could the role of this gene in knee OA progression be related to muscle strength or muscle control? Typically knee OA patients often have effect on symptoms by doing physical therapy increasing their muscle strength and control around the knee?

**Response**:	We have extended our discussion section by discussing the potential role of CDYL2 in more detail. We have rewritten lines 227-231 as follows:

- *“The GTEx eQTL database revealed that one locus near CDYL2, containing six SNPs, showed lower expression of CDYL2 in muscle-skeletal tissue. CDYL2 is involved in regulating genome stability, mitosis, and transcription repression [37, 38]. Given its role in transcription repression, CDYL2 could indirectly influence muscle physiology by modulating gene expression of genes involved in muscle function or development. Moreover, skeletal muscle dysfunction has been implicated as a contributing factor to progression of OA [39]. Additionally, we observed a slightly lower expression of CDYL2 in patients with more decrease in minJSW. This further implies that CDYL2 plays a role in structural knee OA progression, although likely a small role. However, given the lack of studies linking CDYL2 to muscle function or OA progression, this hypothesis remains speculative.”*

You can find the changes in lines 222 – 230 of the revised manuscript.

5) Page 12 line 219: Please remove parenthesis.

**Response**:	We removed the parenthesis. The sentence is now as follows:

- *“We hypothesise that different outcomes, i.e. OA presence or OA progression, and their structural or pain characteristics may have partly or even fully different genetic backgrounds.”*

You can find the changes in lines 218 – 219 of the revised manuscript.

Reviewer #3: The authors have addressed my comments and improved the manuscript now. I suggest the paper to be accepted at its current form.

Reviewer #4: The authors have made significant improvements to the paper and have addressed all my main concerns. I have a few minor comments on the current version:

**Response**:	Thanks for your feedback. Below we respond to your specific remarks and suggestions.

- Italicise gene names on line 37 in abstract

**Response**:	We have italicized the gene names in line 37 in the abstract of the revised manuscript.

- For lines 46-67, I would suggest rephrasing to:
”Despite significant efforts, no effective treatments are available that can delay or even halt cartilage degeneration [2].”

**Response**:	We have adapted the sentence in line 46 as per your suggestion.

- Line 73: add “We calculated a polygenic risk score <(PRS)> using these previously established for OA … “
**Response**:	We have added the abbreviation in line 72 of the revised manuscript.

- Line 109: define “s.d.”
**Response**:	We have defined ‘s.d.’ in line 110 of the revised manuscript.

- Regarding using the IBD cut-off of 0.03125, why was this cutoff selected? This seems quite stringent to me, please justify. How did you choose which one of a related pair to remove? Was this based on genotype missingness?

**Response**:	We have removed samples based on several thresholds. The samples that were removed based on the IBD cut-off were also outliers to the average group heterozygosity and/or had a non-CEU ancestry. Increasing the IBD threshold (to be less stringent) would not alter the results. Therefore, we propose not to change the IBD cut-off.

With the ancestry assessment, what was used to determine “non-European ancestry” (Line 116)? On the PCA plot (Fig S1) I can see two samples grouping with East Asian ancestry, and 11 samples that are admixed European-Asian, which could confound your GWAS. Please justify, and change the axes labels on Fig S1 to PC1 and PC2.
 **Response**:	We have adjusted the axes labels of the PCA plot in the supplementary (Figure S1). Furthermore, the total number of removed samples based on ancestry is indeed 13 (11 mixed European-Asian and 2 East Asian). Some of these 13 samples were also related to another sample or had abnormal heterozygosity and, therefore, were excluded based on multiple QC thresholds. We have now clarified in the figure description that all 13 samples have been excluded from the analysis, and we have adjusted the number in the revised manuscript. You can find this change in line 116 of the revised manuscript.

- Line 118, remove “in”: Previous Genome-wide association studies (GWAS) in large cohorts discovered over 100 OA risk SNPs [7,9]
**Response**:	We have removed ‘in’ in line 119 of the revised manuscript.

- Lines 192-193: Italicise gene names
**Response**:	We have italicised the gene names in line 194 of the revised manuscript.

- Fig S5: Is it possible change this to GRCh37 assembly base positions to be consistent with the rest of the paper? Labelling with rsIDs would help with cross referencing with Table 3.
**Response**:	We have improved Figure S5 by adding the rsIDs in the plot titles. You can find the changes in line 55 of the revised Supplementary.

- Table S1: p-value “0,970” - please change to decimal.
**Response**:	We have replaced the comma by a decimal. You can find this change in line 79 of the revised Supplementary.

- The authors could consider depositing their GWAS results in the GWAS catalog (<https://www.ebi.ac.uk/gwas/>), to assist future research on the same topic.

**Response**:	Thank you for this suggestion. We will definitely consider depositing the GWAS results in this catalog.

Reviewer #5: (No Response)
