# Supplementary material for: Genetic markers for knee osteoarthritis presence are not associated with disease progression - data from the IMI-APPROACH cohort
Source: PLoS One. 2025 Jun 24;20(6):e0325819. doi: 10.1371/journal.pone.0325819 (PMC12186935; doi:10.1371/journal.pone.0325819)
Supplement: Attachment — Submitted filename: Response_to_Reviewers_auresp_3.docx [file pone.0325819.s014.docx]

**Please find a response to the comment of Reviewer #2 below (in blue). Hopefully, we addressed all comments to the intention and satisfaction of the reviewer.**

Reviewers' comments:

Reviewer's Responses to Questions

Comments to the Author

1. If the authors have adequately addressed your comments raised in a previous round of review and you feel that this manuscript is now acceptable for publication, you may indicate that here to bypass the “Comments to the Author” section, enter your conflict of interest statement in the “Confidential to Editor” section, and submit your "Accept" recommendation.

**Reviewer #2:** All comments have been addressed

2. Is the manuscript technically sound, and do the data support the conclusions?

The manuscript must describe a technically sound piece of scientific research with data that supports the conclusions. Experiments must have been conducted rigorously, with appropriate controls, replication, and sample sizes. The conclusions must be drawn appropriately based on the data presented.

**Reviewer #2:** Yes

3. Has the statistical analysis been performed appropriately and rigorously?

**Reviewer #2:** No

4. Have the authors made all data underlying the findings in their manuscript fully available?

The PLOS Data policy requires authors to make all data underlying the findings described in their manuscript fully available without restriction, with rare exception (please refer to the Data Availability Statement in the manuscript PDF file). The data should be provided as part of the manuscript or its supporting information, or deposited to a public repository. For example, in addition to summary statistics, the data points behind means, medians and variance measures should be available. If there are restrictions on publicly sharing data—e.g. participant privacy or use of data from a third party—those must be specified.

**Reviewer #2:** Yes

5. Is the manuscript presented in an intelligible fashion and written in standard English?

PLOS ONE does not copyedit accepted manuscripts, so the language in submitted articles must be clear, correct, and unambiguous. Any typographical or grammatical errors should be corrected at revision, so please note any specific errors here.

**Reviewer #2:** Yes

6. Review Comments to the Author

Please use the space provided to explain your answers to the questions above. You may also include additional comments for the author, including concerns about dual publication, research ethics, or publication ethics. (Please upload your review as an attachment if it exceeds 20,000 characters)

**Reviewer #2:** Thank you for revising the manuscript and addressing all of my comments. However, I still disagree with your choice to include the PRS calculations on the origin sample set.

**Response to the Reviewer #2**:

Thank you for your response. As mentioned in the previous rebuttal letter, we included the PRS calculation on the original sample set in response to a request from another reviewer (Reviewer #4). In the revised manuscript, we further clarified that these results are included for completeness and verification and should not be interpreted as externally validated findings. We updated the relevant text in the Results and Discussion sections accordingly.

Given your statement that “all comments have been addressed,” and the fact that this analysis was included in response to another reviewer’s request, we hope the current manuscript can be accepted. If not, we defer to the Editor’s judgment and are willing to move the PRS results to the Supplementary section.
